# Blocking HIF signaling via novel inhibitors of CA9 and APE1/Ref-1 dramatically affects pancreatic cancer cell survival

**DOI:** 10.1038/s41598-018-32034-9

**Published:** 2018-09-13

**Authors:** Derek P. Logsdon, Fenil Shah, Fabrizio Carta, Claudiu T. Supuran, Malgorzata Kamocka, Max H. Jacobsen, George E. Sandusky, Mark R. Kelley, Melissa L. Fishel

**Affiliations:** 1 Indiana University School of Medicine, Department of Pharmacology and Toxicology, Indianapolis, IN USA; 2 Indiana University School of Medicine, Department of Pediatrics, Wells Center for Pediatric Research, Indianapolis, IN USA; 3 University of Florence, Neurofarba Department, Section of Medicinal Chemistry, Florence, Italy; 4 Indiana University School of Medicine, Department of Nephrology, Indianapolis, IN USA; 5 Indiana University School of Medicine, Department of Pathology and Laboratory Medicine, Indianapolis, IN USA; 6 Indiana University School of Medicine, Department of Biochemistry and Molecular Biology, Indianapolis, IN USA

## Abstract

Pancreatic ductal adenocarcinoma (PDAC) has reactive stroma that promotes tumor signaling, fibrosis, inflammation, and hypoxia, which activates HIF-1α to increase tumor cell metastasis and therapeutic resistance. Carbonic anhydrase IX (CA9) stabilizes intracellular pH following induction by HIF-1α. Redox effector factor-1 (APE1/Ref-1) is a multifunctional protein with redox signaling activity that converts certain oxidized transcription factors to a reduced state, enabling them to upregulate tumor-promoting genes. Our studies evaluate PDAC hypoxia responses and APE1/Ref-1 redox signaling contributions to HIF-1α-mediated CA9 transcription. Our previous studies implicated this pathway in PDAC cell survival under hypoxia. We expand those studies, comparing drug responses using patient-derived PDAC cells displaying differential hypoxic responses in 3D spheroid tumor-stroma models to characterize second generation APE1/Ref-1 redox signaling and CA9 inhibitors. Our data demonstrates that HIF-1α-mediated CA9 induction differs between patient-derived PDAC cells and that APE1/Ref-1 redox inhibition attenuates this induction by decreasing hypoxia-induced HIF-1 DNA binding. Dual-targeting of APE1/Ref-1 and CA9 in 3D spheroids demonstrated that this combination effectively kills PDAC tumor cells displaying drastically different levels of CA9. New APE1/Ref-1 and CA9 inhibitors were significantly more potent alone and in combination, highlighting the potential of combination therapy targeting the APE1-Ref-1 signaling axis with significant clinical potential.

## Introduction

Pancreatic ductal adenocarcinoma (PDAC) is the 4^th^ leading cause of cancer-related death in both men and women in the United States, with an overall five-year survival rate of 8%^1,2^. The therapeutic approaches that have been tested in PDAC have had minimal effects on patient survival^1–3^. The disappointing progress in developing improved treatment strategies for PDAC may be partially explained by the complexity of the tumor-stroma microenvironment over other solid tumors. In addition to the tumor cells, PDAC tumors contain cancer-associated fibroblasts (CAFs), immune cells, and other microenvironment components within a highly reactive stroma, resulting in desmoplastic, hypoxic tumors that are highly aggressive and drug resistant^2–7^.

Hypoxia in PDAC and other tumors is associated with increased growth, invasiveness, and drug resistance^7–9^. Under normal oxygen conditions, Hypoxia-Inducible Factor 1-Alpha (HIF-1α) is rapidly degraded, but decreased oxygen levels lead to its stabilization and dimerization with HIF-1β, resulting in HIF-1-mediated upregulation of factors involved in a variety of tumor-promoting processes^10^. Many indirect methods exist for inhibiting HIF-1α-mediated transcription by targeting HIF-1 transcriptional targets or enzymes involved in regulation of HIF-1 activity, but direct HIF-1-specific inhibitors have not yet been identified^10,11^.

A key subset of HIF-1 transcriptional targets involves pH-regulating enzymes such as carbonic anhydrases (CAs), which help maintain pH homeostasis in cells^12–14^. Of the 16 CAs expressed in human tissue, only CA9 and CA12 are associated with tumors^12,15^. CA9 expression is primarily driven by HIF-1 activity, and it is thought to be a particularly promising therapeutic target in cancer because it is not detected in most normal tissues, but its expression in tumor tissue delineates hypoxic regions and correlates with advanced disease and poor treatment response^13–18^. Several *in vitro* and *in vivo* models have demonstrated the value of targeting CA9 in PDAC cells^19–21^, and a phase I trial evaluating the CA9/12-selective small molecule inhibitor SLC-0111 for safety and tolerability in patients with advanced solid tumors was completed in 2016 (NCT02215850). Moreover, a follow-up trial has been announced that will evaluate SLC-0111 in combination with the PDAC standard-of-care (gemcitabine) in patients with CA9-positive PDAC (NCT03450018).

In addition to O_2_ regulation of HIF-1α, HIF-1 transcriptional activity is increased by redox signaling via Apurinic/Apyrimidinic Endonuclease-1-Reduction/oxidation Effector Factor 1 (APE1/Ref-1)^15,22–24^. APE1/Ref-1 was initially discovered as a DNA endonuclease in Base Excision Repair (BER), but it was later found to play an important role in redox signaling via reduction of oxidized cysteine residues in specific transcription factors (TFs) to modulate their transcriptional activity^24–26^. APE1/Ref-1 redox signaling regulates the activity of several TFs, notably HIF-1α, as well as STAT3 and NFκB^24^. APE1/Ref-1 expression is a biomarker for poor prognosis in patients with solid tumors, and its importance in cancer has been validated in numerous pre-clinical models of a wide array of tumor types^15,24–26^. The small molecule APX3330 (formerly E3330) is a direct APE1/Ref-1 inhibitor that is highly selective for APE1/Ref-1 redox signaling activity without affecting APE1/Ref-1 endonuclease activity in tumor cells^24,27–29^. Its safety and tolerability have been validated in both animal and human studies^22,24,30,31^, but an ongoing clinical trial (NCT03375086) will establish its tolerability and appropriate dosing in patients with solid tumors, including PDAC, for future phase II trials.

APE1/Ref-1 redox signaling promotes CA9 expression via HIF-1-mediated transcription, as evidenced by the reduction of hypoxia-induced expression of CA9 following APE1/Ref-1 knockdown or inhibition with APX3330^15^. We also previously demonstrated enhanced killing of hypoxic PDAC cells and 3D PDAC tumor spheroids with the combination of APX3330 and SLC-0111^15^. Therefore, this project centers on continuing that work with more potent, second-generation drug analogs and further dissecting the specific effects of these combination treatments on tumor cells. Novel APX3330 analogs have been developed that have improved potency as APE1/Ref-1 redox signaling inhibitors *in vitro*, namely APX2009 and APX2014 (Patent 9,089,605)^24,32–34^. Additionally, an analog of SLC-0111 and novel CA9 inhibitor, FC12-531A shows improved potency over SLC-0111 and enters cells to additionally inhibit cytosolic CAs^35–37^.

This work evaluates differential hypoxia signaling between PDAC cell lines and the consequent differences in CA9-targeting drug response. We also provide evidence linking APE1/Ref-1 redox signaling directly to HIF-1 DNA binding in hypoxic cells and expand upon our previous finding that dual-targeting APE1/Ref-1 redox signaling and CA9 activity leads to enhanced tumor cell killing in a 3D PDAC tumor model. The significant interest in each of these enzymes as targets in cancer therapy makes this project highly relevant for potential future clinical application. Moreover, the use of second-generation agents that are still in development extends the potential relevance of this project well into the future by focusing on these targets as part of a targeted, precision oncology approach for PDAC patients with these altered pathways.

## Materials and Methods

### Cell Culture

Low-passage patient-derived PDAC cell lines (fewer than 35 passages from patient tumors) and pancreatic cancer-associated fibroblasts used in this project were received from Dr. Anirban Maitra (The Johns Hopkins University) and maintained as previously described^15,22,38,39^. Cells were submitted for STR analysis (CellCheck with IDEXX BioResearch) and were regularly confirmed to be free of mycoplasma contamination. Cell lines were passaged fewer than 12 times before resuscitating fresh stocks. Hypoxic conditions in monolayer experiments were generated in a Ruskinn Invivo_2_ 200 hypoxia work station (Baker Ruskinn; Sanford, ME) at 0.2% oxygen. Cell proliferation and viability in monolayer cultures was measured with alamarBlue assay as previously described^15,39^. Growth of 3-dimensional tumor spheroid cultures was performed and quantified as described previously using tumor cells stably transduced with TdTomato (red), and CAFs stably transduced with EGFP (green) to differentiate the two cell types in 3D co-cultures and track the growth of each over time^15,40,41^.

### Western Blot Analysis

Immunoblotting was performed as previously described^15,39^ with antibodies for APE1/Ref-1 (Novus Biologicals; Littleton, CO), CA9 (Santa Cruz; Dallas, Texas), Actin (NeoMarkers; Fremont, CA), and Vinculin (Sigma; St. Louis, MO). All samples were processed and run in parallel.

### siRNA Transfections

PDAC cells were transfected with siRNA as previously described^15,38,39^. siRNAs used were scrambled control and siAPE1/Ref-1 (previously reported)^15,38,39^, as well as OriGene (Rockville, MD) Trisilencer siCA9. For specific sequences of the siRNAs, please see Supplemental Table 1.

### Inhibitors

Small molecule inhibitors were prepared and used as previously described^15,22,38,39^. APE1/Ref-1 redox signaling was inhibited using APX3330, APX2009, and APX2014 (Apexian Pharmaceuticals; Indianapolis, IN)^15,22,38,39^. RN7–58 (Apexian Pharmaceuticals) was used as a negative control that, although structurally similar, does not inhibit APE1/Ref-1 redox signaling activity^24,32–34,39^. CA9 inhibition was accomplished with SLC-0111^15,17,35,36,42^ and FC12-531A^35–37^. For more information and compound structures, see Supplemental Table 2.

### ChIP Assay

Chromatin Immunoprecipitation (ChIP) was performed using the Magna ChIP kit (Millipore). Immunoprecipitation (IP) was performed using polyclonal antibodies for HIF-1α (Novus) or Rabbit IgG control (Millipore). Binding to the HIF-1-Binding Site (HBS) in the CA9 promoter was measured via qPCR using SYBR Green master mix (Applied Biosystems) in a CFX96 Real-Time System (Bio-Rad). Primer sequences used for ChIP qPCR were: CA9 HBS-Fwd (5′-CTCACTCCACCCCCATCCTA-3′) and CA9 HBS-Rev (3′-GATCAACAGAGGGAGCCAGG-5′). The amplicon length was 249 bp. Melt curves were carried out to confirm single peaks, and amplified products were run on 1% agarose gels to confirm single bands. 1% of the cross-linked DNA from each sample was evaluated (without IP) as a control to normalize the qPCR signal across samples (Input).

### pH Assay

Intracellular pH was measured with pHrodo Red AM Intracellular pH Indicator (LifeTech) as described^15^. Fluorescent images of pHrodo Red AM-exposed cells were captured with a confocal/two-photon Olympus Fluoview FV-1000 MPE system (Olympus Scientific Solutions America; Waltham, MA) at the Indiana Center for Biological Microscopy facility (Indianapolis, IN) as previously described^15^. Imaging was performed by Dr. Malgorzata Kamocka, who was blinded to the treatments in each well.

### qRT-PCR

mRNA levels were measured using qRT-PCR as previously described^15,22,39^. The comparative C_t_ method was used to quantitate mRNA levels using RPLP0 (ribosomal protein lateral stalk subunit P0) and B2M (Beta-2-Microglobulin) as reference genes^43,44^. The primers for CA9, RPLP0, and B2M are commercially available (Applied Biosystems). Experiments were performed in triplicate for each sample.

### Immunohistochemistry (IHC)

3D spheroid cultures were collected on day 12 after plating, fixed with 4% paraformaldehyde (PFA) (Electron Microscopy Sciences; Hatfield, PA), and permeabilized with 70% ethanol. Fixed/permeabilized 3D cultures were solidified in HistoGel (LifeTech). HistoGel plugs were paraffin embedded and slides were prepared by the laboratory of Dr. Keith Condon (Indiana University School of Medicine; Indianapolis, IN). Samples were stained with the specified antibodies by the Indiana University School of Medicine Research Immunohistochemistry Facility (Indianapolis, IN) and quantified using the HALO image analysis platform (Indica Labs) as before^45^.

### Statistical analysis

Comparisons in experiments with more than two groups were analyzed with post-hoc Multiple Comparisons Tests (Tukey, Dunnett, or Sidak as appropriate)^15,46,47^. Differences between groups were considered significant if p < 0.05. Statistical analyses were performed with Microsoft Excel and Prism (Version 6.0f, Copyright ©2014 GraphPad Software Inc. La Jolla, CA).

## Results

### Importance of the APE1/Ref-1-HIF-1-CA9 signaling axis in PDAC cells

**Figure 1:**
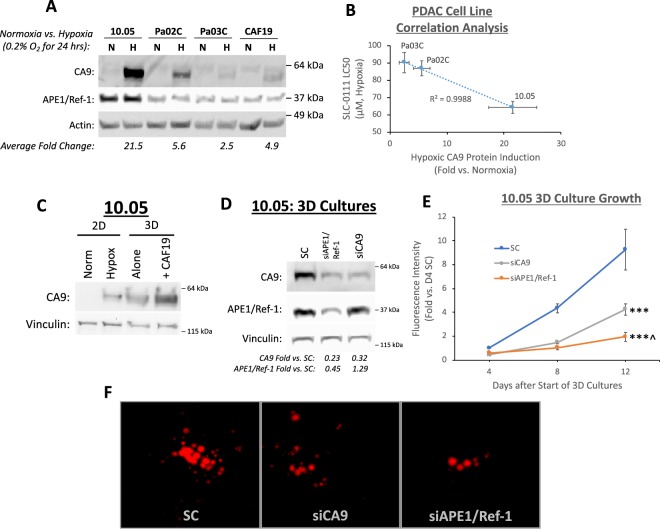
Hypoxia-induced CA9 expression and constitutive APE1/Ref-1 expression are important for 3D PDAC tumor spheroid growth. (**A**) Patient-derived PDAC tumor cell lines 10.05, Pa02C, and Pa03C, as well as the pancreatic CAF cell line CAF19, were exposed to 0.2% oxygen for 24 h, and CA9 protein levels were compared via western blot (p < 0.05 for all cell line differences between normoxia and hypoxia). Representative blot of N = 3. (**B**) LC50 values for SLC-0111 in PDAC cell lines under hypoxic conditions (0.2% O2) are inversely correlated with CA9 induction in each cell line (R2 > 0.99, N = 3). (**C**) Comparison of CA9 protein induction between 10.05 cells grown in monolayer (2D, 0.2% O2, 24 h) and in 3D cultures alone or in co-culture with CAF19 cells for 12 days. N = 1 (**D**–**F**). 10.05 Cells were transfected with the indicated siRNAs and cultured in 3D spheroids. Cells were collected for western blot analysis on Day 8 to confirm knock-down (**D**, p < 0.05 for siAPE1/Ref-1 and siCA9 effects on CA9 as well as siAPE1/Ref-1 effects on APE1/Ref-1, N = 3). Fluorescence intensity (**E**) of both tumor (TdTomato) and CAFs (EGFP) was measured to track 3D tumor growth on days 4, 8, and 12 of 3D cultures (+/−SEM, ***p < 0.001 for differences between knockdown groups and SC on D12, ^^^p < 0.05 for difference between siAPE1/Ref-1 and siCA9 on D12, N = 3). Representative fluorescent images from each group were captured on day 12 (**F**). Full size blots are shown in Supplemental Figs S3–S5.

In order to fully characterize the response of different PDAC patient lines to hypoxia, the relative CA9 expression in low-passage patient-derived PDAC cell lines (10.05, Pa02C, and Pa03C) was determined following 24 h. exposure to 0.2% oxygen, and compared to cells incubated in normoxic conditions. Although CA9 is well-established as a hypoxia-regulated enzyme, the level of CA9 expression induced under hypoxic conditions is variable between patient lines. Hypoxia exposure significantly induced CA9 protein levels in all cell lines (2.5–21.5-fold), including cancer-associated fibroblast cells (CAF19) (Fig. 1A)^15^. However, CA9 was most strongly induced in the 10.05 cells (21.5-fold over normoxia), and Pa03C cells had the lowest levels of hypoxia-induced CA9 protein (2.5-fold over normoxia). Notably, APE1/Ref-1 expression was not significantly affected by hypoxia exposure in these cells. Although APE1/Ref-1 expression was strongest in the cell line with the strongest CA9 expression (10.05), no significant differences in APE1/Ref-1 expression were observed in the remaining cell lines, indicating that the differences in CA9 expression between these cell lines are not likely to be primarily driven by differences in APE1/Ref-1 levels. The majority of mechanistic experiments were evaluated in 10.05 cells because of the robust CA9 induction in these cells, but key experiments were repeated in the Pa03C cells to confirm and compare responses to the treatment strategy investigated here.

The efficacy of SLC-0111, a CA9 inhibitor that has recently completed a Phase I clinical trial, was evaluated in different PDAC cell lines under hypoxic conditions. While all three of the patient-derived PDAC cell lines tested had IC_50_s for SLC-0111 below 100 μM during hypoxia exposure (Fig. 1B), the most sensitive cell line was 10.05, which induces CA9 expression to the highest level following hypoxia exposure (Fig. 1A). In fact, CA9 protein induction under hypoxia was inversely correlated with SLC-0111 IC_50_ under hypoxia in the cell lines tested (R^2^ > 0.99) (Fig. 1B), indicating that increased CA9 induction may predict increased sensitivity to CA9 inhibition in PDAC cells. We therefore hypothesized that blocking CA9 production would increase tumor cells’ reliance on the available CA9 under hypoxic conditions, effectively sensitizing tumor cells to CA9 inhibition especially under hypoxia.

We also compared the induction of CA9 in pancreatic cancer cells grown in monolayer versus 3D tumor spheroids. Following exposure to 0.2% hypoxia using a hypoxic chamber in the 10.05 monolayer cultures, CA9 was induced ~21-fold (Fig. 1A,C). Using the 3D tumor spheroid system with and without CAFs, CA9 expression was further increased 2.5 and 6.2-fold, respectively, over hypoxic monolayer cultures despite these spheroid cultures being grown in normoxic conditions (Fig. 1C). This indicates the 3D tumor systems contain more physiologic hypoxic signaling and are more robust models for predicting patient tumor responses^15,40,41,48–53^. Moreover, the increased CA9 expression in tumor +CAF co-cultures vs. tumor-only 3D cultures indicates that the CAF cells are likely contributing to an increase in the size of the spheroids and further signaling complexity that lead to increased hypoxia signaling in the tumor cells. Differences in exposure between Fig. 1A,C indicate that exogenous hypoxia and 3D culture-induced hypoxia are similar or even more intense in 3D culture. Based on the relevance of the 3D culture model, the effects of blocking HIF signaling via inhibition of CA9 and APE1/Ref-1 were further evaluated in this system.

Knockdown of APE1/Ref-1 or CA9 expression was evaluated in 3D spheroid cultures to demonstrate that the growth and survival of PDAC cells in this relevant microenvironment was dependent upon CA9 and APE1/Ref-1 expression. Spheroids were collected on day 8 to confirm the continued knockdown of CA9 and APE1/Ref-1. As expected, each siRNA significantly reduced the expression of its target gene/enzyme product, confirming the continued knockdown of these enzymes throughout the duration of the 3D cultures. Remarkably, APE1/Ref-1 knockdown also decreased CA9 expression similarly to that seen in siCA9 cultures (Fig. 1D). Reduced levels of APE1/Ref-1 and/or CA9 significantly inhibited 3D tumor spheroid growth by ~50–80% (p < 0.001) as measured by fluorescence intensity and area (Fig. 1E,F). These results confirm the importance of APE1/Ref-1 and CA9 expression in PDAC tumor growth. Additionally, APE1/Ref-1 knockdown inhibited 3D tumor spheroid growth significantly more than CA9 knockdown, which suggests that inhibition of CA9 alone may not be as efficient at attenuating PDAC tumor growth as dual-targeting these enzymes.

### Blockade of CA9 via APE1/Ref-1 or CA9 inhibition

**Figure 2:**
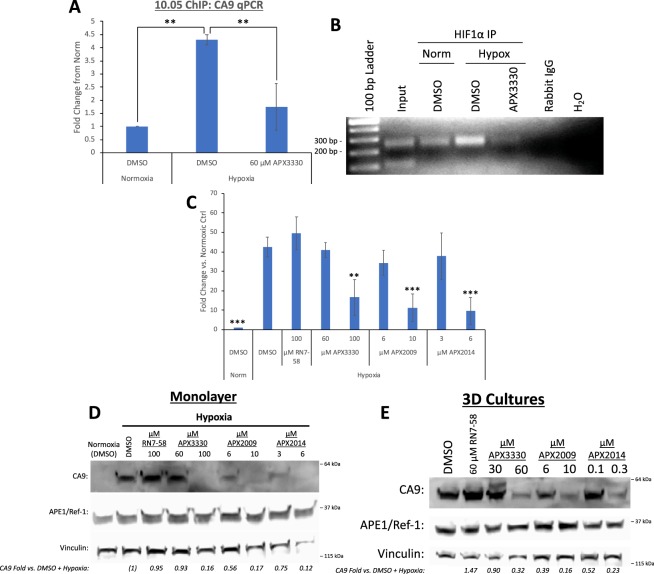
APE1/Ref-1 redox signaling inhibition decreases HIF-1-mediated CA9 expression in PDAC cells. (**A**,**B**) 10.05 cells were treated with APX3330 and exposed to 0.2% O2 for 12 h prior to protein-DNA cross-linking and collection. IPs of HIF1a and a control for non- specific binding were performed using nuclear extracts. qPCR for the HBS-containing region of the CA9 promoter was performed. (**A**, **p < 0.01, N = 3). PCR for the HBS-containing region of the CA9 promoter was performed using IP samples as well as input DNA (1% of amount loaded into IPs − performed on DMSO + Hypoxia sample) and a negative control (H2O), (**B**, expected product size = 249 bp). (**C**,**D**) 10.05 cells were treated with APX3330, APX2009, APX2014, and the negative analog RN7-58 and exposed to 0.2% O2 for 24 h prior to collection and analysis of CA9 mRNA (**C**, N = 4) and protein (**D**, N = 3) levels (p < 0.01 for differences in CA9 mRNA and protein levels at the highest concentration tested of each APE1/Ref-1 inhibitor vs. DMSO). (**E**) 10.05 cells were cultured in 3D spheroids for 12 days prior to collection and Western Blot analysis. Cultures were treated with the indicated concentrations of APX3330, APX2009, APX2014, and the negative analog RN7-58 on days 4 and 8 (p < 0.05 for differences in CA9 expression at the highest concentration tested of each APE1/Ref-1 inhibitor vs. DMSO, N = 3–4). Full size blots are shown in Supplemental Figs S6 and S7.

Inhibition of APE1/Ref-1 redox signaling via APE1/Ref-1 siRNA decreases HIF-1-mediated CA9 transcription^15,22,23^ and subsequent expression (Fig. 1D). We hypothesized that this was the result of a direct effect on HIF-1α binding to the CA9 promoter, so a chromatin immunoprecipitation (ChIP) assay was performed to investigate HIF-1-DNA interactions. 10.05 cells were treated with the APE1/Ref-1 redox signaling inhibitor APX3330 and exposed to 0.2% O_2_ for 12 h. Immunoprecipitations (IPs) of HIF-1α demonstrated a 4.3-fold increase in HIF-1α binding to the HIF-1-Binding Site (HBS) in the CA9 promoter under hypoxic conditions, and this effect was decreased by ~60% in cells treated with APX3330 (Fig. 2A,B). These data demonstrate that HIF-1α interactions with the CA9 promoter are induced by hypoxia, and that these hypoxia-induced interactions are attenuated by APE1/Ref-1 redox signaling inhibition, providing further confirmation of the mechanism by which APE1/Ref-1 redox signaling regulates HIF-1-mediated CA9 transcription under hypoxic conditions.

Our previous work demonstrated that the combination of APE1/Ref-1 and CA9 inhibition was more effective than either drug alone, but in the micromolar range^15^. In order to build upon this regimen and demonstrate target selectivity, we used two analogs of APX3330, APX2009 and APX2014 (Patent 9,089,605)^24,32–34^, and an analog of SLC-0111, FC12-531A, all of which demonstrate improved cytotoxicity over their parent compounds under hypoxic conditions (Table 1).

**Table 1:** IC_50_ values for PDAC lines in Monolayer and 3D culture.

			10.05	Pa03C
Monolayer (Hypoxia)		APX3330	40.3	50.2
		APX2009	4.9	6.7
		APX2014	1.3	3.5
		SLC-0111	63.9	90.8
		FC12-531A	1.4	1.1
3D Cultures	Tumor Alone	APX3330	44.5	37.9
		APX2009	9.4	6.9
		APX2014	0.3	1.1
		SLC-0111	64.0	51.9
		FC12-531A	4.8	3.0
	Tumor + CAFs: Tumor Cells	APX3330	57.9	41.6
		APX2009	13.0	8.4
		APX2014	1.2	2.4
		SLC-0111	70.8	64.1
		FC12-531A	4.9	3.8
	Tumor + CAFs: CAF Cells	APX3330	59.4	57.3
		APX2009	14.1	13.1
		APX2014	6.3	7.1
		SLC-0111	98.2	97.0
		FC12-531A	4.6	5.3

*N = 3.

**All concentrations are µM.

***3D culture data calculated from D12 curves.

Hypoxia-induced CA9 mRNA and protein levels were evaluated in 10.05 cells following treatment with APX3330, APX2009, APX2014, and the inactive analog RN7-58. Inhibition of APE1/Ref-1 with all three inhibitors resulted in concentration-dependent decreases in hypoxia-induced CA9 mRNA (Fig. 2C) and protein levels (Fig. 2D), with 10-fold less required for APX2009 and APX2014 vs. APX3330, indicating that these analogs are more potent inhibitors of APE1/Ref-1 redox signaling than APX3330. The inactive analog (RN7-58) did not affect hypoxia-induced CA9 expression even at 100 μM, confirming the APE1/Ref-1 redox signaling function in HIF1-induced CA9 expression and the specificity and on target effects of the APX compounds for APE1/Ref-1.

The effect of APE1/Ref-1 redox inhibition on CA9 protein expression was subsequently measured in 3D tu mor spheroids. 10.05 cells were grown in 3D cultures for 12 days and treated on days 4 and 8 with the APX compounds or the inactive analog (RN7-58). APE1/Ref-1 redox inhibition significantly decreased the expression of CA9 protein in 3D tumor cultures in a concentration-dependent manner (Fig. 2E). Importantly, the more potent APE1/Ref-1 inhibitors once again required 10-fold less of the concentration compared to APX3330 to attenuate CA9 expression in the spheroid cultures, further validating the potency of these newer compounds and having efficacy at sub-uM doses. The inactive analog (RN7-58) did not affect CA9 expression in PDAC spheroid cultures, once again corroborating the specificity of the effects of the APE1/Ref-1 redox signaling inhibitors on HIF-1α activity.

**Figure 3:**
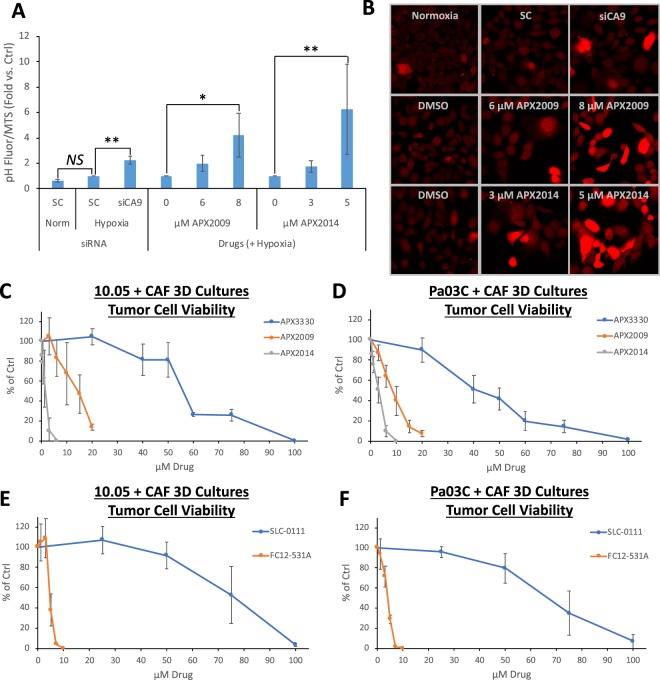
Blockade of CA9 via APE1/Ref-1 or CA9 inhibition acidifies and kills PDAC cells. (**A**,**B**) 10.05 cells were transfected with the indicated siRNAs or treated with the indicated concentrations of APX2009 or APX2014 and exposed to 0.2% O2 for 48 h (N = 3). Images of pH-mediated fluorescence changes are shown in (**B**). (**C**–**F**) 3D Co-cultures with 10.05 (**C**,**E**) or Pa03C (**D**,**F**) tumor cells (+CAFs) were treated with increasing concentrations of APX3330, APX2009, and APX2014 or SLC-0111 and FC12-531A for 12 days, and fluorescence intensity was measured (N = 3–6 +/−SD, normalized to DMSO ctrl).

CA9 expression is important for tumor cell growth, and CA9 functions by stabilizing intracellular pH to counteract the acidification that occurs in response to metabolic changes under hypoxic conditions. As a functional marker for CA9 activity, the effects of CA9 on intracellular pH were evaluated. Hypoxia exposure (0.2% O_2_ for 48 h) did not significantly affect intracellular pH in 10.05 cells (Fig. 3A,B), indicating that these cells compensate for the effects of hypoxia on pH. However, when CA9 expression was reduced via siRNA, the result was a significant decrease in intracellular pH in hypoxia-exposed cells, as measured by increased fluorescence of the ph-sensitive pHrodo Red AM dye (Fig. 3A,B). These data support the conclusion that CA9 is responsible for regulating pH in these cells.

As previously reported, APX3330 and SLC-0111 did not affect intracellular pH as single-agents at concentrations up to 50 μM APX3330 or 100 μM SLC-0111, instead requiring the combination of both compounds to acidify hypoxic PDAC cells^15^. Therefore, the APX3330 analogs, APX2009 and APX2014, were used to determine whether APE1/Ref-1 redox inhibition alone can shift intracellular pH in hypoxic PDAC cells given a sufficiently potent inhibitor. Treatment with either 8 μM APX2009 or 5 μM APX2014 alone resulted in a significant increase in fluorescence (normalized to cell survival), indicating decreased intracellular pH with single-agent APE1/Ref-1 redox signaling inhibition (Fig. 3A,B). These data demonstrate that APE1/Ref-1 redox signaling inhibition alone is sufficient to acidify cells as expected due to the decrease in CA9 expression following APE1/Ref-1 inhibition. Taken together, the data in Fig. 3A,B indicate that decreases in CA9 expression via either siRNA or APE1/Ref-1 redox inhibition results in intracellular acidification in hypoxic PDAC cells, further supporting APE1/Ref-1 mechanism of action (MOA).

Following these promising mechanistic results, the effects of APX2009 and APX2014 were compared to APX3330 in 3D PDAC tumor +CAF co-cultures using 10.05 (Fig. 3C) or Pa03C (Fig. 3D) tumor cells. Each of these analogs exhibited IC_50_s between 4-50-fold lower than APX3330 in this 3D tumor spheroid model (Table 1), suggesting that they have improved potency in their tumor growth-inhibitory effects compared to APX3330. Similarly, a more potent analog of CA9 inhibitor, SLC-0111, FC12-531A exhibited IC_50_s ~15-fold lower than SLC-0111 in this 3D tumor spheroid model (Fig. 3E,F and Table 1), demonstrating improved potency in its tumor growth-inhibitory effects over SLC-0111. Interestingly, while SLC-0111 was a more potent inhibitor of tumor cells than CAFs in the 3D co-culture system, many of the drugs tested had similar IC50s for both CAFs and tumor cells in this model, especially in co-culture spheroids containing 10.05 cells (Table 1). However, concentrations below these IC50s were identified for each drug that affected tumor cell growth/survival without significant killing in the CAFs and are used in subsequent combination experiments involving dual inhibition of APE1/Ref-1 and CA9. This system underscores the importance of evaluating tumor-stroma interactions following drug treatment and the differential response between different tumor cells as well as CAFs.

### Characterization of 3D cultures and effects of dual-targeting APE1/Ref-1 and CA9

**Figure 4:**
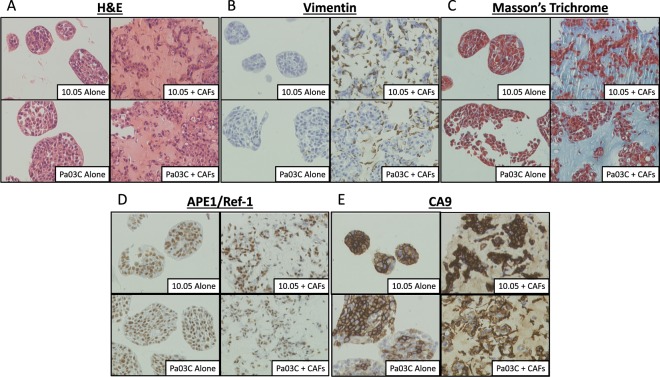
Characterization and effect of CAFs on 3D cultures. Spheroids consisting of 10.05 (Top) or Pa03C (Bottom) cells were cultured alone (left) or with CAFs (right) for 12 days and collected for IHC. Antibody stains (**B**,**D**,**E**) were counter-stained with hematoxylin. Images are 1,600x magnification. N = 3.

Characterization of 3D spheroid cultures was performed using H&E staining, Vimentin (to detect CAFs within the co-cultures), and Masson’s Trichrome (Fig. 4A–C). This staining shows that the tumor cells are more densely packed than the CAFs in the 3D co-cultures as expected, and the Masson’s Trichrome staining indicates that the CAFs predominantly deposit extracellular matrix (ECM) components such as collagen, again validating this model as representative of the fibrotic tumors seen in PDAC patients (Fig. 4A–C). Notably, APE1/Ref-1 staining is primarily nuclear and appears to be uniform in tumor cells throughout the cultures in all 3D cultures tested (Fig. 4D). Furthermore, the hypoxia marker CA9 show positivity in distinct regions (Fig. 4E), suggesting differential zones of hypoxia within the spheroid cultures. These data demonstrate that culturing PDAC cells in this 3D tumor spheroid model results in hypoxic cells that express CA9 without the need for external induction of low-oxygen conditions and support the use of these 3D spheroid co-culture conditions to more accurately simulate physiological conditions. Similar to monolayer in Fig. 1A, CA9 expression was significantly lower in Pa03C spheroids (tumor cells only, ~55% positive cells) vs. spheroids consisting of 10.05 cells alone (~97% positive cells) (p < 0.001). However, CA9 expression in the co-culture spheroids grown in the presence of CAFs was not significantly different between 10.05 and Pa03C cultures, indicating that co-culture conditions promote hypoxia signaling, and therefore CA9 expression, similarly in both tumor cell types (Figs 4E and S1A).

**Figure 5:**
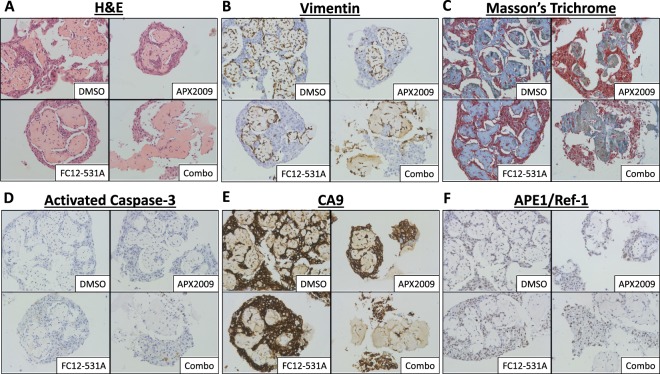
Effects of dual-targeting APE1/Ref-1 and CA9 on activated Caspase-3, CA9, and APE1/Ref-1 expression. 10.05 +CAF spheroid co-cultures were collected on Day 12 after treatment with Vehicle (DMSO), 10 µM APX2009, 3 µM FC12-531A, or both drugs together. Antibody stains (**B**,**D**–**F**) were counter-stained with hematoxylin. Images are 400x magnification. N = 3.

In order to evaluate the consequences of APE1/Ref-1 and CA9 inhibition on various markers of cellular functions, 3D tumor +CAF co-cultures consisting of 10.05 and CAF19 cells were treated with 10 μM APX2009 and/or 3 μM FC12-531A on Days 4 and 8, then collected for IHC on Day 12 (Fig. 5). Treatment with APX2009 alone decreased both the number of Vimentin positive cells and the area of fibrosis detected via Masson’s Trichrome by ~70% (p < 0.001), but these effects returned to baseline in spheroids treated with the combination of APX2009 and FC12-531A (Figs 5B,C and S1B,D). Meanwhile, combination treatment with APX2009 and FC12-531A resulted in a ~55% decrease in tumor cell number/density without affecting ECM area (Figs 5A–C and S1B–D), suggesting that this combination treatment strategy is effective at selectively targeting tumor cells over CAFs. Interestingly, each drug alone decreased the percentage of cells that were Vimentin positive, but the combination of both inhibitors increased this percentage by ~14% (p < 0.001) (Figs 5B and S1C), providing further evidence that the combination of APE1/Ref-1 redox inhibition and CA9 inhibition results in preferential killing of tumor cells as opposed to CAF cells in this 3D co-culture system. IHC for activated Caspase-3 was used as a marker for apoptosis in these 3D co-culture spheroids. Neither drug had a significant effect on activated Caspase-3 positivity alone at this time point, but the combination of APX2009 and FC12-531A significantly increased the proportion of remaining cells that were positive for activated Caspase-3 from 0.3% in DMSO to 0.9% (p < 0.01) (Figs 5G and S1H), providing confirmation that the observed decrease in tumor cell number/density with this combination treatment strategy is at least partially due to cell killing.

Although APX2009 at 10 μM was sufficient to decrease CA9 expression in tumor alone 3D spheroids (without CAFs) (Fig. 2E), this concentration of APX2009 did not affect CA9 positivity in tumor +CAF co-culture spheroids (Figs 5E and S1F), suggesting that CAF cells are protective against the effects of APE1/Ref-1 redox inhibition in these cultures. However, the combination of APX2009 and FC12-531A in the co-culture spheroids decreased the proportion of cells that were positive for CA9 by ~8% (p < 0.01) (Figs 5E and S1F). Given the results described above showing preferential killing of tumor cells with this drug combination, it is likely that this decrease in CA9 positivity results from a shift in the tumor-to-CAF ratio within these spheroid co-cultures following dual-targeting of APE1/Ref-1 and CA9. Although none of these treatments resulted in a significant change in APE1/Ref-1 positivity, a non-significant trend was observed with a slight decrease in the proportion of APE1/Ref-1-positive cells following combination treatment with APX2009 and FC12-531A (Figs 5D and S1E), which may also be attributable to the shift in tumor:CAF ratios with this combination treatment strategy.

### APE1/Ref-1 redox signaling inhibition sensitizes 3D PDAC tumor spheroids to CA9 inhibition with second-generation inhibitors

We previously demonstrated in 3D co-cultures containing both PDAC tumor cells and CAFs that combination targeting of APE1/Ref-1 and CA9 significantly attenuated tumor cell growth with minimal effects on the CAFs in the spheroid co-cultures at Day 12 of co-culture^15^. To characterize the growth inhibitory effects of the compounds over time and determine the optimal regimen, we extended the time in culture to 16 days and assayed for growth after each treatment (Fig. S2A–F). Again, the combination therapy was more effective than monotherapy in both patient-derived cell lines. We also combined SLC-0111 with APX2009 at 10 μM or APX2014 at 0.6 μM in 3D co-cultures, resulting in similar trends to those seen with APX3330 at 50 μM (Fig. S2G–R).

**Figure 6:**
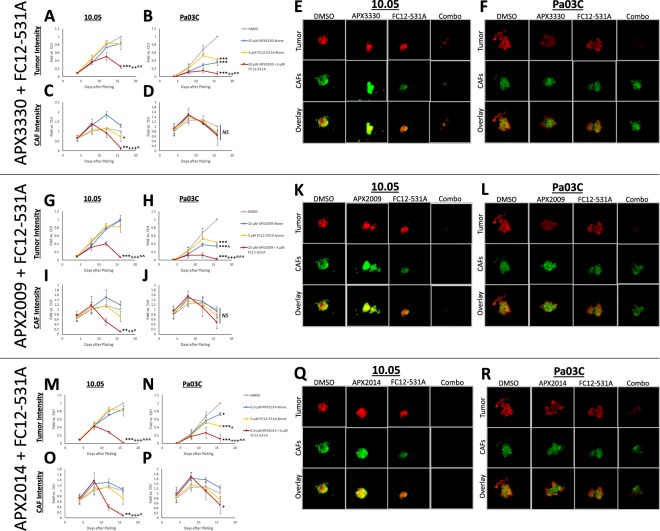
APE1/Ref-1 redox signaling inhibition dramatically sensitizes 3D PDAC tumor spheroids to CA9 inhibition with second-generation inhibitors. 10.05 and Pa03C cells were plated into 3D cultures with CAF19 cells, and size of the spheroids was measured via fluorescence intensity on days 4, 8, 12, and 16 after plating. 3D cultures were treated with FC12-531A + APX3330 (**A**–**F**), +APX2009 (**G**–**L**), or + APX2014 (**M**–**R**) following measurements on days 4, 8, and 12. Fluorescence intensity data within each experiment were normalized to day 16 DMSO ctrl, and day 16 readings were compared. Differences between groups were determined using Tukey’s multiple comparisons test: *p < 0.05 vs. DMSO; **p < 0.01 vs. DMSO; ***p < 0.001 vs. DMSO; ^+^p < 0.05 vs. APE1/Ref-1 Inhibitor; ^++^p < 0.01 vs. APE1/Ref-1 Inhibitor; ^+++^p < 0.001 vs. APE1/Ref-1 Inhibitor; ^^^p < 0.05 vs. FC12-531A; ^^^^p < 0.01 vs. FC12-531A; ^^^^^p < 0.001 vs. FC12-531A. Graphs are means with standard deviations of N = 3. Fluorescent images of Tumor (Red) and CAF (Green) cells in these spheroids were captured on day 16.

To further evaluate the effects of CA9 inhibition and APE1/Ref-1 inhibition in PDAC cells, we employed the SLC-0111 analog, FC12-531A, which yielded similar results to SLC-0111 in the tumor cells in this model, but with substantially lower concentrations needed to achieve similar effects on tumor growth inhibition (3 μM FC12-531A vs. 50 μM SLC-0111). Specifically, FC12-531A monotherapy (3 μM) significantly affected tumor cell growth in Pa03C co-cultures, but not in 10.05 co-cultures, and FC12-531A significantly enhanced the effects of APX3330, APX2009, and APX2014 on 3D tumor cell growth in both 10.05 and Pa03C co-cultures (Fig. 6). These data validate our previous results showing enhanced PDAC tumor cell killing with dual-targeting of APE1/Ref-1 redox signaling and CA9 activity. Additionally, these results demonstrate that the new analogs have an improved potency in the nanomolar-to-low-micromolar concentrations of each drug with the most potent combination (0.6 μM APX2014 + 3 μM FC12-531A, Fig. 6M–R). Interestingly, FC12-531A affected CAF growth in 10.05 3D co-cultures, but not in Pa03C 3D co-cultures, and APE1/Ref-1 redox inhibition enhanced the killing effects of FC12-531A on CAFs, especially in 10.05 3D co-cultures. This may indicate a role for intracellular CAs in the CAF effects since FC12-531A is more lipophilic than SLC-0111^35–37^, and these data further suggest that the tumor – CAF interactions are also patient-specific.

## Discussion

**Figure 7:**
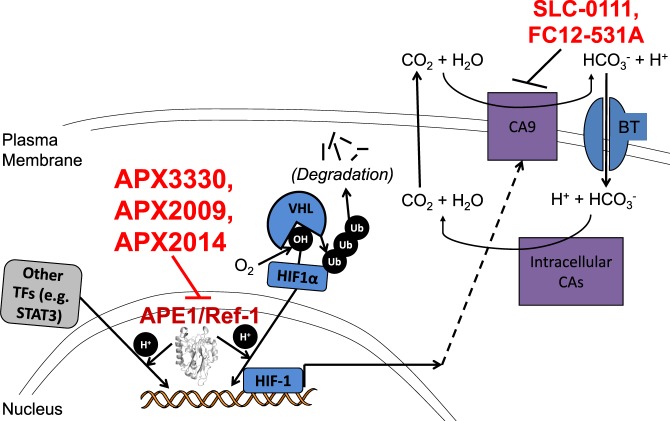
Pathway schematic. APE1/Ref-1 redox signaling contributes to the transactivation of HIF-1 and certain other transcription factors. HIF1α is stabilized under hypoxic conditions, leading to the formation of HIF-1 and subsequent expression of CA9. CA9 coordinates with the bicarbonate transporter and intracellular CAs to stabilize intracellular pH. APE1/Ref-1 redox signaling inhibition (with APX3330, APX2009, or APX2014) attenuates HIF-1-mediated CA9 expression, sensitizing tumor cells to CA9 inhibition (with SLC-0111 or FC12-531A).

Our findings evaluate the APE1/Ref-1 node connecting APE1/Ref-1 redox signaling through HIF-1-mediated transcription to CA9 expression and activity (Fig. 7). The data presented here expands the therapeutic significance of this signaling axis using novel analogs of clinical compounds to effectively block the vital APE1/Ref-1-HIF-1-CA9 pathway. The blockade of these two targets created a “drug-synthetic-lethality”, resulting in enhanced killing of tumor cells in spheroid co-cultures^15,24,54^. These data strongly support targeting the DNA binding of HIF-1 and other key transcription factors via redox inhibition of APE1/Ref-1 in combination with blockade of another aspect of HIF-1 signaling in order to more effectively shut down the HIF pathway, resulting in increased tumor cell death. By inhibiting the redox function of APE1/Ref-1 in combination with CA9 inhibition, we propose that the compensatory response is more effectively blocked in PDAC cells compared to inhibition of either target alone. Our studies demonstrated for the first time that hypoxia-induced interactions between HIF-1 and the promoter of one of its major transcriptional targets, CA9, are decreased following APE1/Ref-1 redox signaling inhibition (Fig. 1C). These data provide further mechanistic understanding of APE1/Ref-1 contributions to HIF-1-mediated transcription. Given the role of CA9 in regulating tumor cell pH, which affects overall survival and cellular function, the findings elucidated here contribute to hypoxia signaling and pH regulation in PDAC cells with significant translational implications.

APE1/Ref-1 redox activity and CA9 activity are important for tumor cell growth and survival, and CA9 functions as part of the pH regulatory cycle in hypoxic cells^13,14,16–18,24–26,49^. Therefore, the effects of this signaling axis on intracellular pH were evaluated as a functional marker for CA9 activity. We previously published that neither 50 μM APX3330 nor 100 μM SLC-0111 affected intracellular pH alone, but that the combination of both inhibitors resulted in a significant decrease in intracellular pH^15^. Here we present data comparing knockdown of CA9 with siRNA to treatment with APX2009 or APX2014 at concentrations sufficient to decrease CA9 expression and show that intracellular pH is decreased in both cases (Fig. 3A,B). These data support the conclusion that CA9 operates in these cells by regulating pH and that APE1/Ref-1 redox signaling inhibition alone is sufficient to acidify cells via decreased CA9 expression given a sufficiently potent inhibitor.

Recent work from our laboratory used single cell RNA sequencing (scRNA-Seq) to identify genes that were differentially expressed following APE1/Ref-1 knockdown in primary PDAC cells and examined the signaling pathways affected by APE1/Ref-1^55^. The unbiased scRNA-Seq results confirm data presented here, demonstrating that HIF-1 signaling was significantly downregulated following APE1/Ref-1 knockdown^55^. CA9 mRNA levels in the RNA-Seq analysis were also significantly downregulated following APE1/Ref-1 knockdown despite the cells being fully oxygenated for this analysis. We have observed this effect using qPCR analysis in some cell lines^15^, suggesting that some baseline HIF-1 transcriptional activity occurs under normoxic conditions and that this activity is still under APE1/Ref-1 redox control.

The use of multiple patient-derived tumor cell lines and the differential response between the various patient lines underscores a potential precision oncology approach in targeting the APE1/Ref-1-HIF-CA9 pathway for PDAC treatment. Specifically, we demonstrate in Fig. 1A,B that CA9 induction under hypoxic conditions is different among the primary PDAC cell lines evaluated, and that these differences correlate with sensitivity to the CA9 parent compound inhibitor, SLC-0111. Evaluation of clinical data may determine the usefulness of CA9 expression in tumor tissue as a biomarker for predicting the utility of SLC-0111. This is supported by the design of the current clinical trial evaluating SLC-0111 in combination with gemcitabine (NCT03450018), which will evaluate this combination in patients with CA9-positive PDAC. However, the cell-permeable analog FC12-531A exhibited no correlation between CA9 expression and drug sensitivity, indicating that inhibition of other CAs may be useful in patients whose tumors have low levels of CA9 induction. Furthermore, the combination of inhibition of HIF-1 through APE1/Ref-1, coupled with CA9 function inhibition was effective in both cases (high and low CA9 induction), indicating that this strategy may be valuable in a wider range of cases than inhibition of CA9 alone. Still, clinical trials using any of these compounds should evaluate patients with CA9-positive tumors separately from CA9-negative patients to further tease apart the potential differences in response between these groups.

In addition to monolayer cell culture experiments, we employed state-of-the-art 3D tumor spheroid co-cultures to further evaluate APE1/Ref-1 and CA9 targeting in a more robust model system with physiologic hypoxic regions, collagen deposition, and more accurate portrayals of patient tumor behavior^15,40,41,48–52^. In fact, recent evidence suggests that *in vivo* patient-derived xenograft (PDX) models undergo rapid genomic shifts, taking on the copy-number alterations of their host mice as they are passaged, indicating that low-passage, patient-derived *ex vivo* models such as this spheroid co-culture system actually may retain more of the original tumor’s genetic characteristics than PDX *in vivo* models^53^. Interestingly, APE1/Ref-1 redox signaling inhibition resulted in a greater enhancement of the effects of CA9 inhibition in the 3D co-culture model than was seen in monolayer cultures under either normoxic or hypoxic conditions (data not shown), which highlights the value of the more robust 3D co-culture model.

Likewise, the 3D co-culture model allows for measurements of the effects of the dual-treatment strategy employed here on both tumor cells and CAFs as they interact in a dynamic system with differential zones of hypoxia and drug accessibility. The CAF cells in the 3D co-cultures displayed differing sensitivities to the compounds tested depending on the tumor cells present, highlighting the importance of studying patient-to-patient variability in tumor-stroma interactions as they relate to treatment effects. This is particularly significant because stromal destruction in established PDAC tumors has been implicated in hastening metastatic spread^3,5,56^, while “reprogramming” stromal cells into a quiescent state has shown promise as an alternate approach to block stromal contributions to tumor growth/spread without ablating the stromal “restraint” of tumors^2,3,56–58^. Our IHC data following APE1/Ref-1 inhibition with APX2009 demonstrates that blocking APE1/Ref-1 signaling in the CAFs has significant effects on the vimentin-positivity and the deposition of collagen by the CAFs (Figs 5B,C and S1B,D), and yet is not killing them as seen in Fig. 6I,J. This suggests a reprogramming of the CAFs following treatment that we plan to further investigate in future studies.

The results presented in this manuscript support a dual-targeting strategy with second-generation APE1/Ref-1 inhibitors and CA9 inhibitors resulting in enhanced inhibition of 3D PDAC tumor growth. Both APX3330 and SLC-0111 have exhibited minimal *in vivo* toxicity in numerous pre-clinical experiments, showing promise for advancement to phase II trials^13,15,17–19,21,24^. Future clinical trials could therefore evaluate the efficacy of the strategy presented here in patients with solid tumors. Moreover, this work evaluates novel analogs of APX3330 and SLC-0111 as part of efforts to develop a pipeline of additional inhibitors that may have even larger therapeutic windows than the existing APE1/Ref-1 or CA9 inhibitors. To that end, these analogs have also been administered in mice with minimal toxicity, though ongoing studies are evaluating their safety and efficacy alone and in combination with other agents *in vivo*. The data with APX2009, APX2014, and FC12-531A provide further evidence of the importance of targeting APE1/Ref-1 redox signaling and CA activity in tumor cells and confirm the on-target effects of the existing drugs. Additionally, since APE1/Ref-1 and CA9 are elevated in a variety of other solid tumors, this combination treatment will likely have applicability beyond pancreatic cancer^16,17,24^. In conclusion, the results in this work provide additional exploration of the close relationship between APE1/Ref-1, HIF-1, and CA9 in PDAC as well as evidence supporting the combination of two small molecule inhibitors (and their pipelines of analogs), each exhibiting minimal toxicity, that may help treat patients with PDAC, a deadly disease that has persistently evaded treatment.

## Electronic supplementary material

Supplemental information and figures

## Data Availability

All datasets generated as part of this study are available from the corresponding author upon reasonable request.
