# Supplementary material for: Blocking HIF signaling via novel inhibitors of CA9 and APE1/Ref-1 dramatically affects pancreatic cancer cell survival
Source: Sci Rep. 2018 Sep 13;8:13759. doi: 10.1038/s41598-018-32034-9 (PMC6137035; doi:10.1038/s41598-018-32034-9)

Supplementary Information

Blocking HIF signaling via novel inhibitors of CA9 and APE1/Ref-1 dramatically affects pancreatic cancer cell survival

Derek P. Logsdon, Fenil Shah, Fabrizio Carta, Claudiu T. Supuran, Malgorzata Kamocka, Max H. Jacobsen, George E. Sandusky, Mark R. Kelley^, Melissa L. Fishel*^

*Corresponding author; ^ Co-last authors.

Supplemental Table 1: List and target sequence of siRNA sequences used in the knockdown experiments.

Supplemental Table 2: Structures of Ref-1 and CA9 inhibitors used in these studies.

Supplemental Figure 1: Quantification of 3D culture IHC. A. The percentage of cells that were positive for CA9 expression in 10.05 (blue) or Pa03C (orange) spheroids grown alone (left) or with CAF19 cells was quantified as described in methods. B-H. 10.05 + CAF spheroid co-cultures were collected on Day 12 after treatment with Vehicle (DMSO), 10 µM APX2009, 3 µM FC12-531A, or both drugs together. The total number (B) and percentage (C) of cells that were positive for Vimentin expression was quantified. The total area of blue staining in Masson's Trichrome was quantified (D). The percentage of cells that were positive for activated caspase-3 (E), CA9 (F), and APE1/Ref-1 (G) was quantified. Representative images are found in Figures 4 & 5. *p<0.05, **P<0.01, ***p<0.001

Supplemental Figure 2: APE1/Ref-1 redox signaling inhibition sensitizes 3D PDAC tumor spheroids to CA9 inhibition. 10.05 and Pa03C cells were plated into 3D cultures with CAF19 cells, and cell growth in these spheroids was measured via fluorescence intensity on days 4, 8, 12, and 16 after plating. 3D cultures were treated with SLC-0111 + APX3330 (A-F), + APX2009 (G-L), or + APX2014 (M-R) following measurements on days 4, 8, and 12. Fluorescence intensity data within each experiment were normalized to day 16 DMSO control, and day 16 readings were compared. Differences between groups were determined using Tukey's multiple comparisons test: *p<0.05 vs. DMSO; **p<0.01 vs. DMSO; ***p<0.001 vs. DMSO; +p<0.05 vs. APE1/Ref-1 Inhibitor; ++p<0.01 vs. APE1/Ref-1 Inhibitor; +++p<0.001 vs. APE1/Ref-1 Inhibitor; ^p<0.05 vs. SLC-0111;^^p<0.01 vs. SLC-0111; ^^^p<0.001 vs. SLC-0111. Graphs are means with standard deviations of N = 3. Fluorescent images of Tumor (Red) and CAF (Green) cells in these spheroids were captured on day 16.

Supplemental Figure S3 – S7: Full blots for the Western blots shown in the manuscript.

Supplemental Table 1

siRNA		Target Sequence
APE1/Ref-1	#1	GTCTGGTACGACTGGAGTA
	#2 (LifeTech # s1445)	GATTAGATTGGGTAAAGGA
	#3 (LifeTech # s1446)	CAGATATACTGTGCCTTCA
	#4 (LifeTech # s1447)	ACCCTATGCCTACACCTTT
CA9 (OriGene Cat # SR300537)	A	GCTGTCTCGCTTGGAAGAAATCGCT
	B	ATAATGCCCACAGGGACAAAGAAGG
	C	AGAGGGCTCCCTGAAGTTAGAGGAT
CA12 (OriGene Cat # SR300539)	A	AGATGTCAAATCGTGGTTTAGATCA
	B	TGCATCATCAGATGACAAGGACAAC
	C	CCATTATAACTCAGACCTTTATCCT

Supplemental Table 2

Name	Compound Structure	M.W.	Known Targets
APX3330	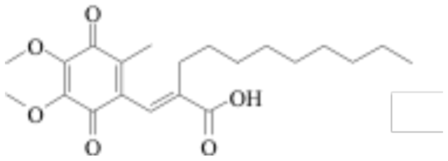	378.5	APE1/Ref-1
APX2009	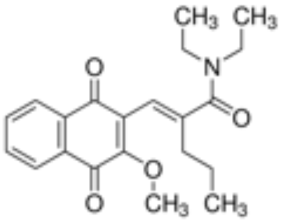	355.4	APE1/Ref-1
APX2014	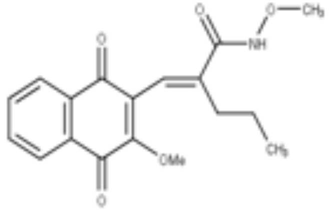	343.4	APE1/Ref-1
RN7-58	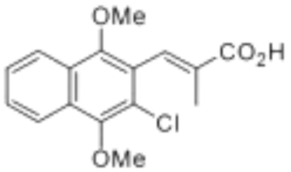	306.7	N/A (Inactive Analog of APX compounds)
SLC-0111	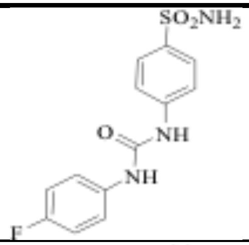	309.3	CA12 (Ki = 5 nM), CA9 (Ki = 45 nM), CA4/CA14 (Ki's = 250-450 nM), CA2 (Ki = 960 nM), other cytosolic CA's (Ki's > 1 μM)
FC12-531A	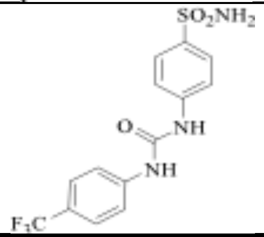	359.1	CA12 (Ki = 2.3 nM), CA9 (Ki = 6.2 nM), CA1 (Ki = 9.7 nM), CA2 (Ki = 1,150 nM), other CA's (Ki's unknown)

Supplemental Figure S1.

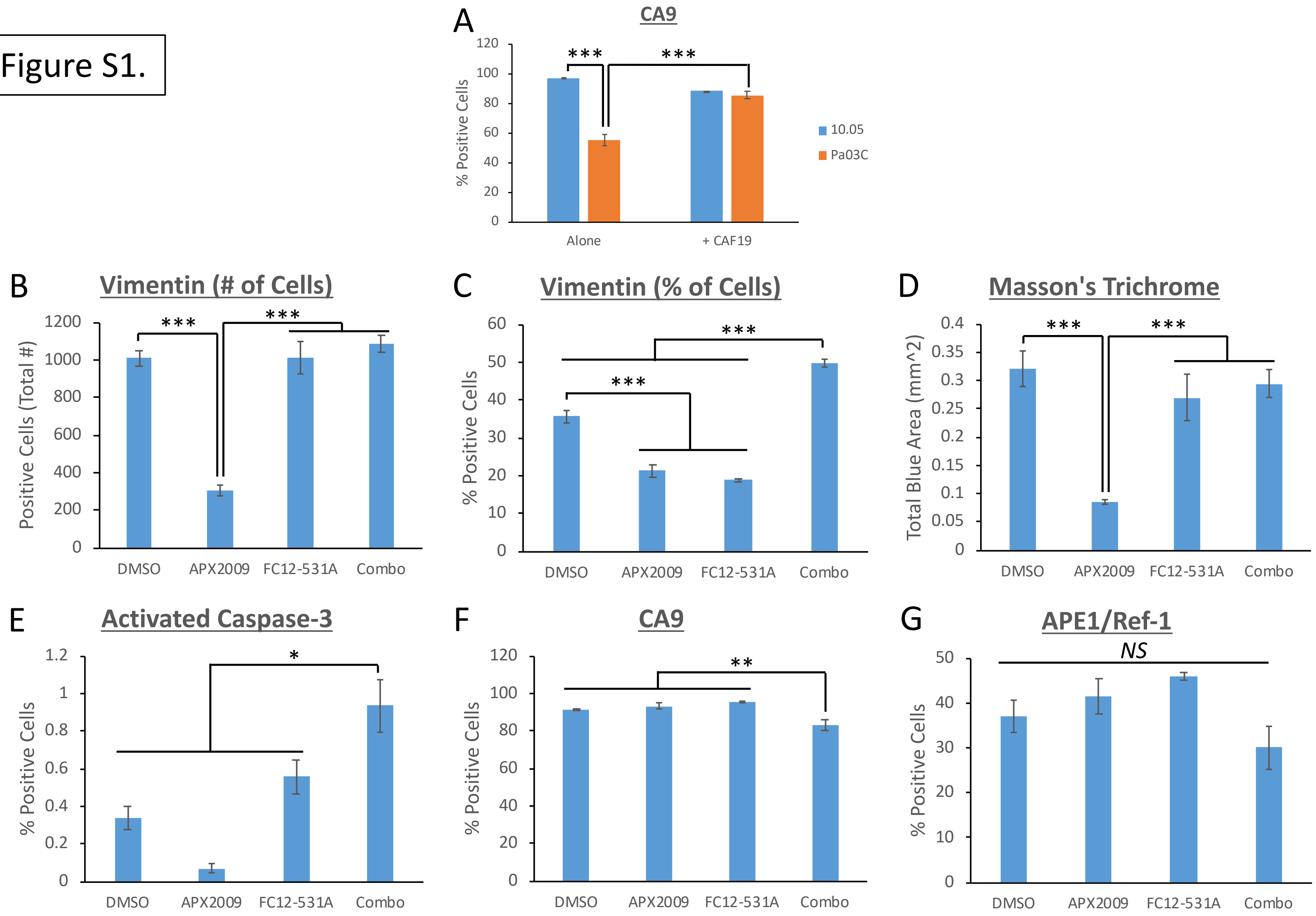

Supplemental Figure S2.

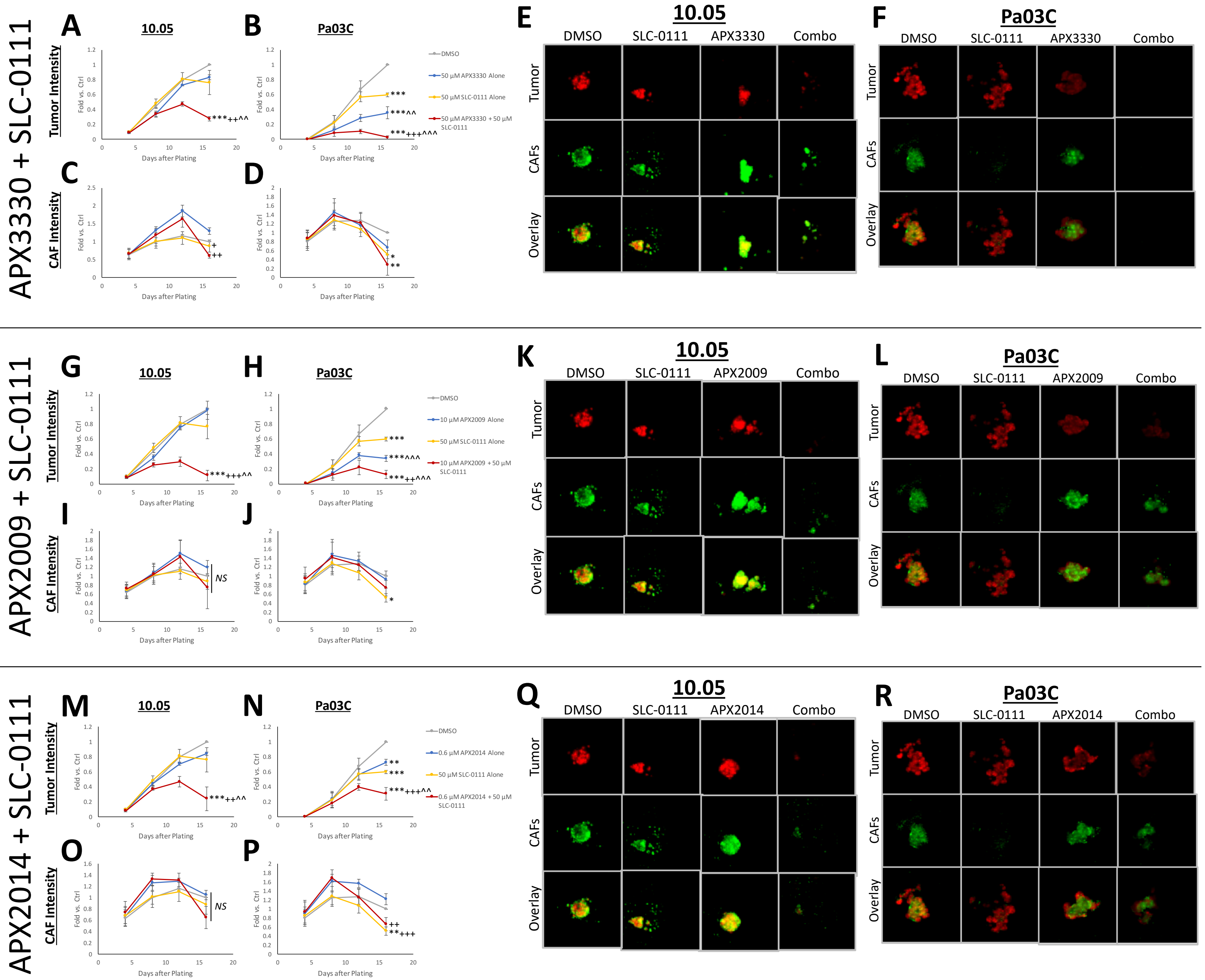

Supplemental Figure S3A. Full blot for Figure 1A: CA9

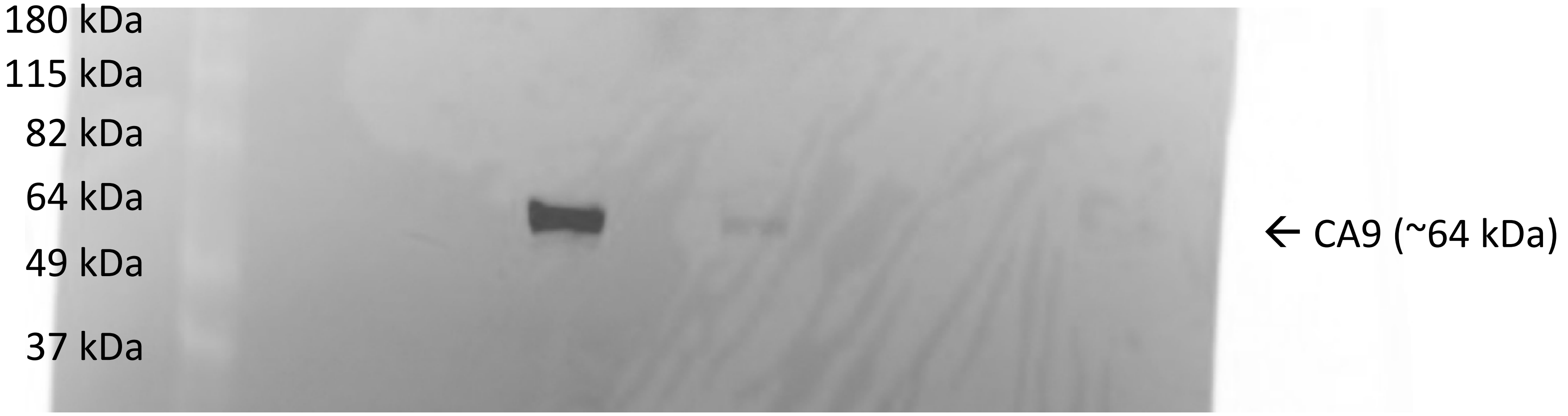

Supplemental Figure S3B. Full blot for Figure 1A: APE1/Ref-1

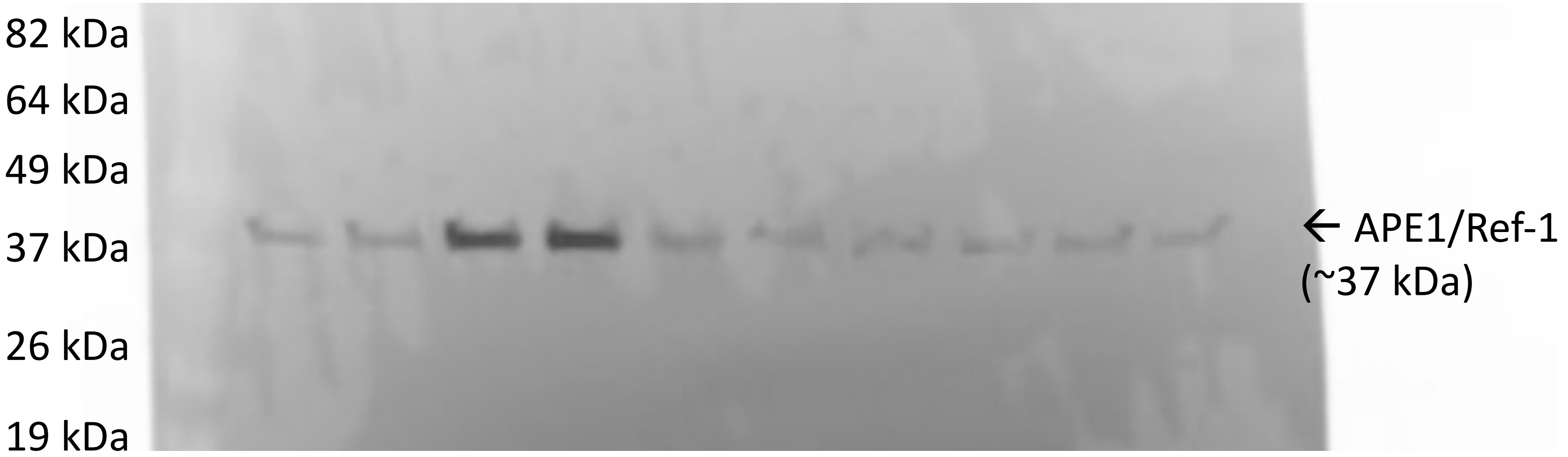

Supplemental Figure S3C. Full blot for Figure 1A: Actin

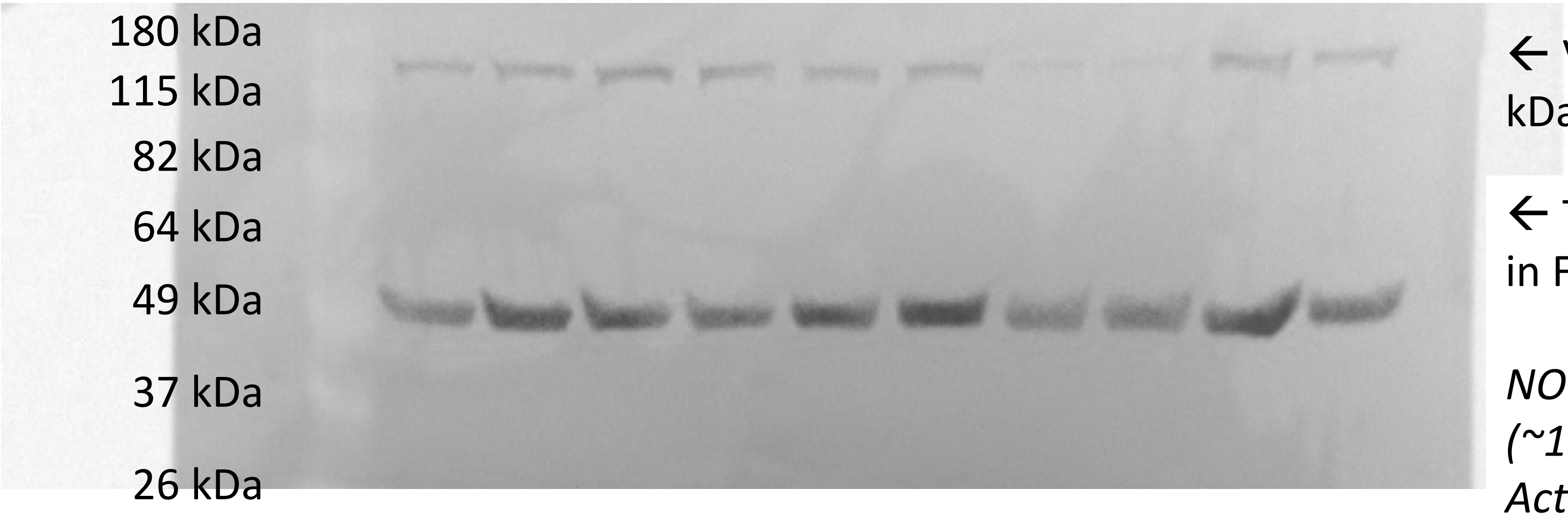

← Vinculin (~130 kDa)

← Top Blot used in Figure 1A

NOTE: Vinculin (~130 kDa) & Actin (~50 kDa) probed simultaneously

Supplemental Figure S4A. Full blot for Figure 1C: CA9

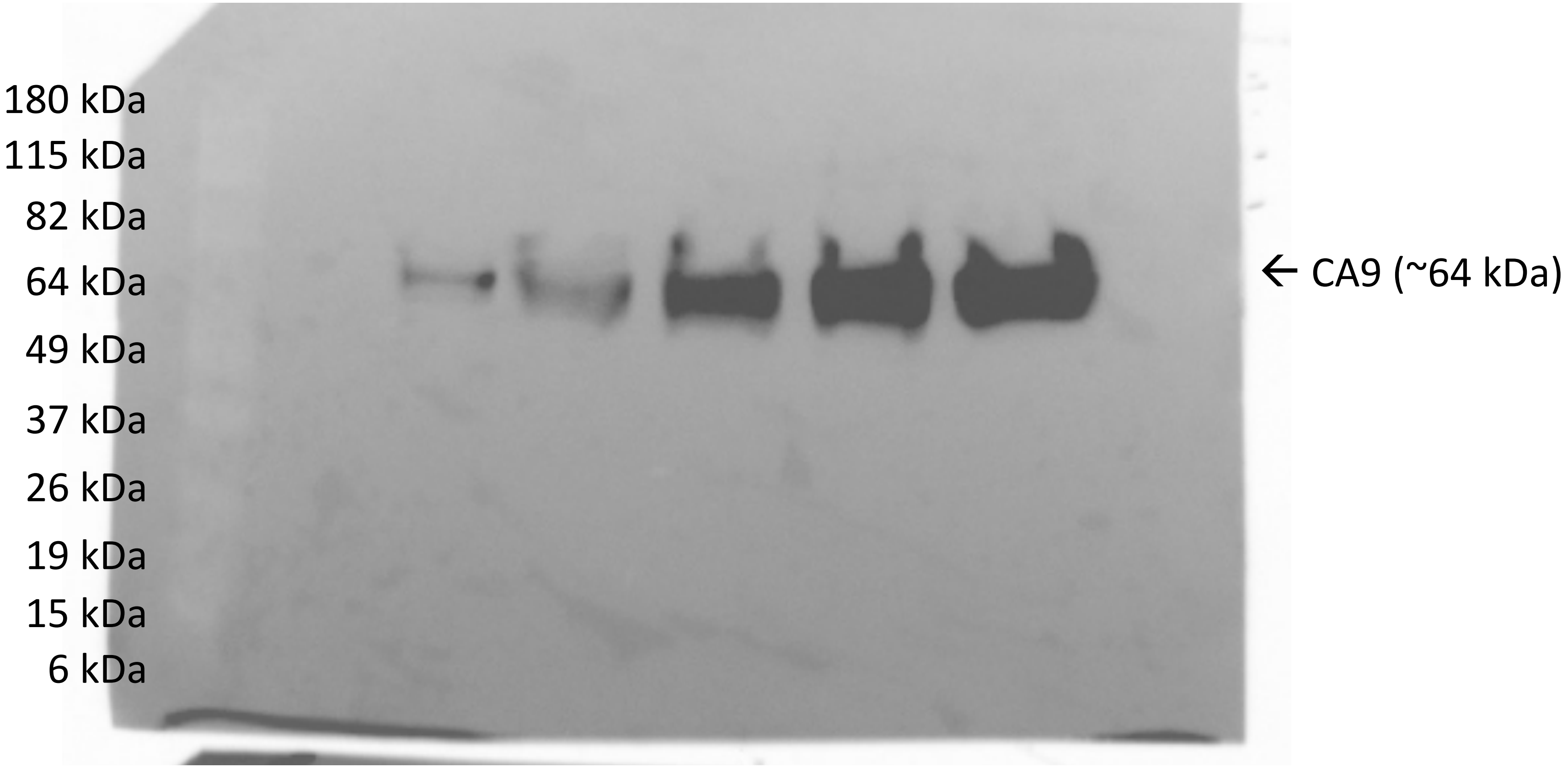

Supplemental Figure S4B. Full blot for Figure 1C: Vinculin

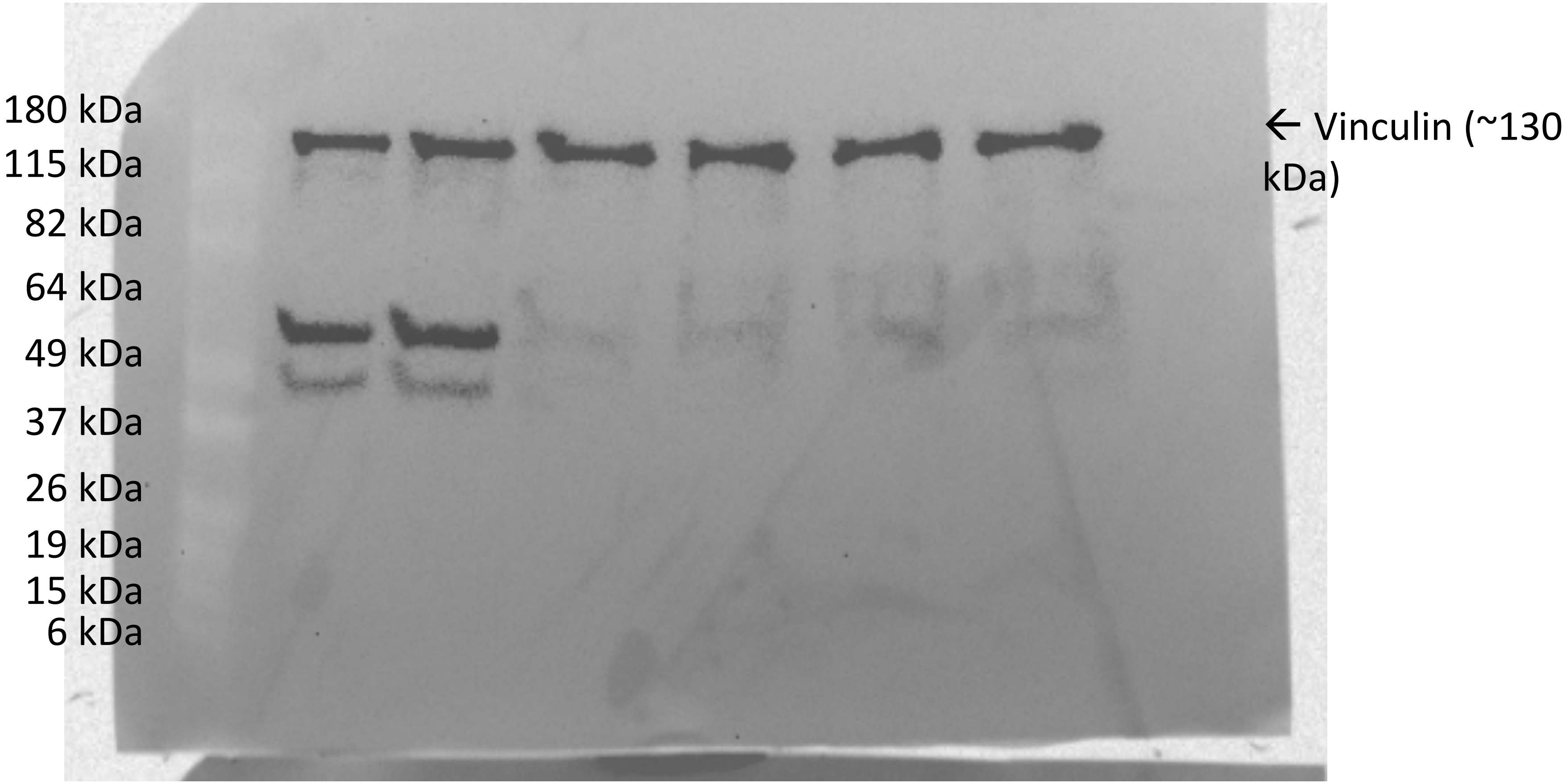

NOTE: Vinculin (~130 kDa) & Actin (~42 kDa) probed simultaneously

Supplemental Figure S5A. Full blot for Figure 1D: CA9

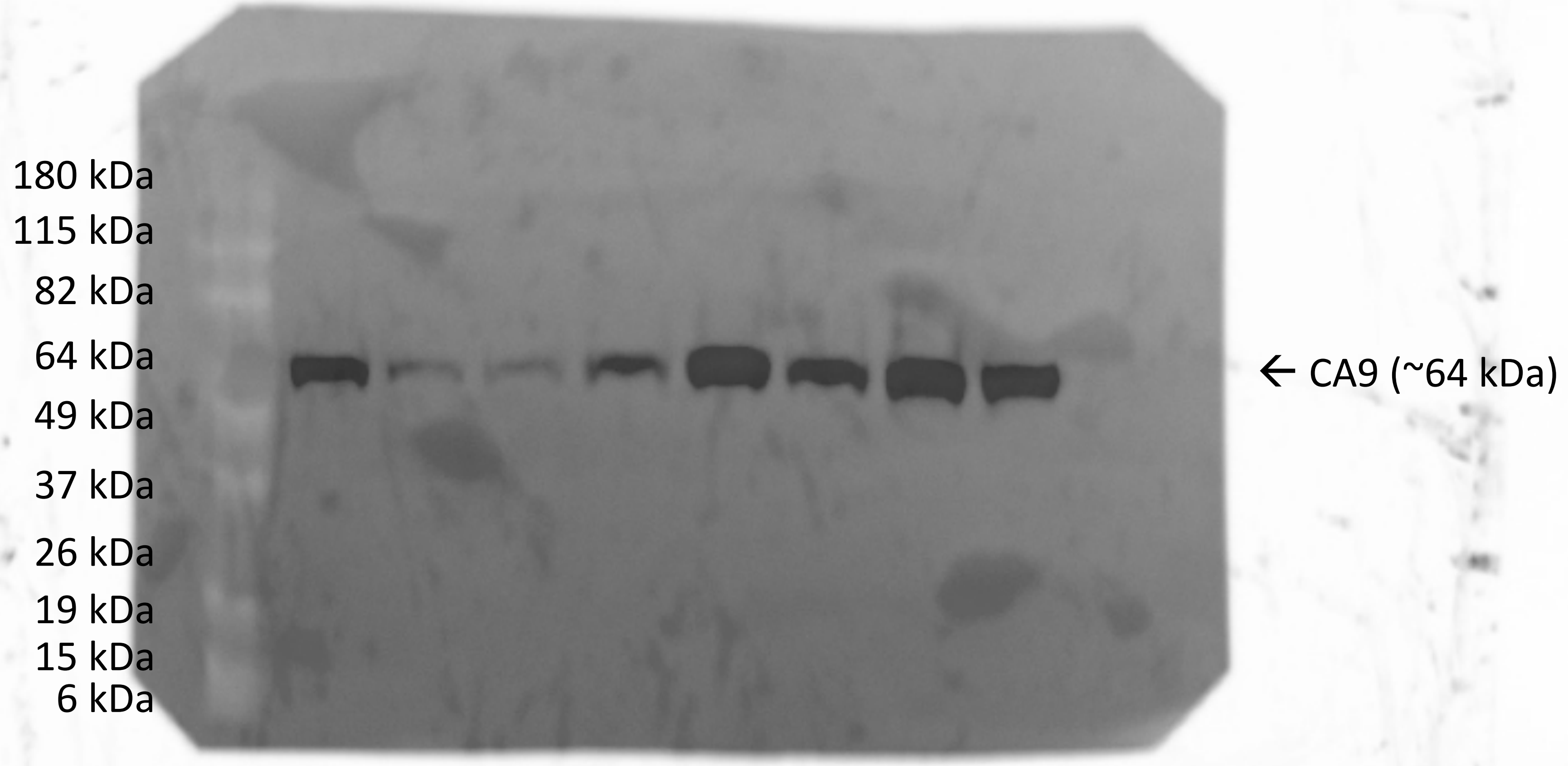

Supplemental Figure S5B. Full blot for Figure 1D: APE1/Ref-1

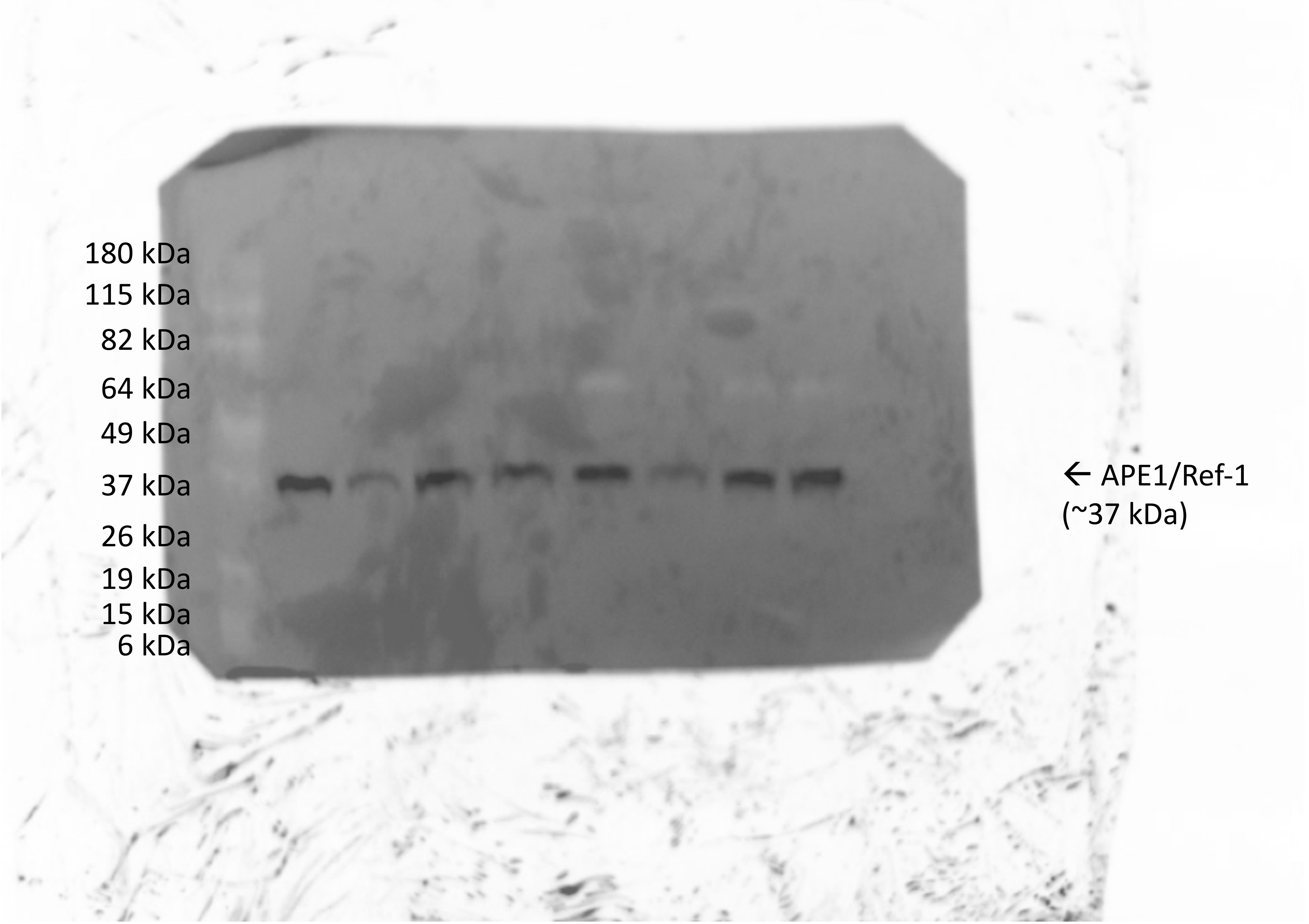

Supplemental Figure S5C. Full blot for Figure 1D: Vinculin

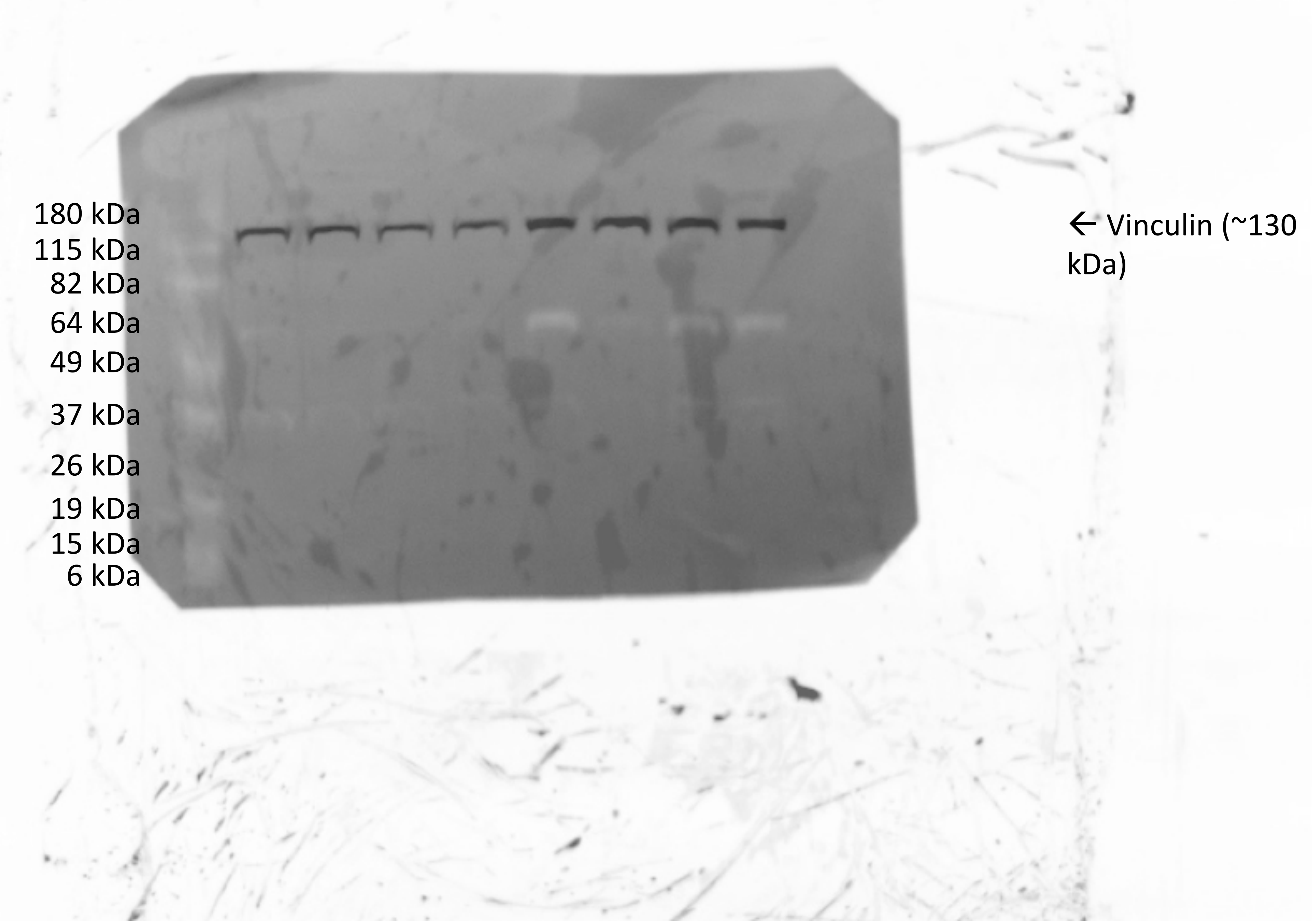

Supplemental Figure S6A. Full gel for Figure 2B: ChIP assay

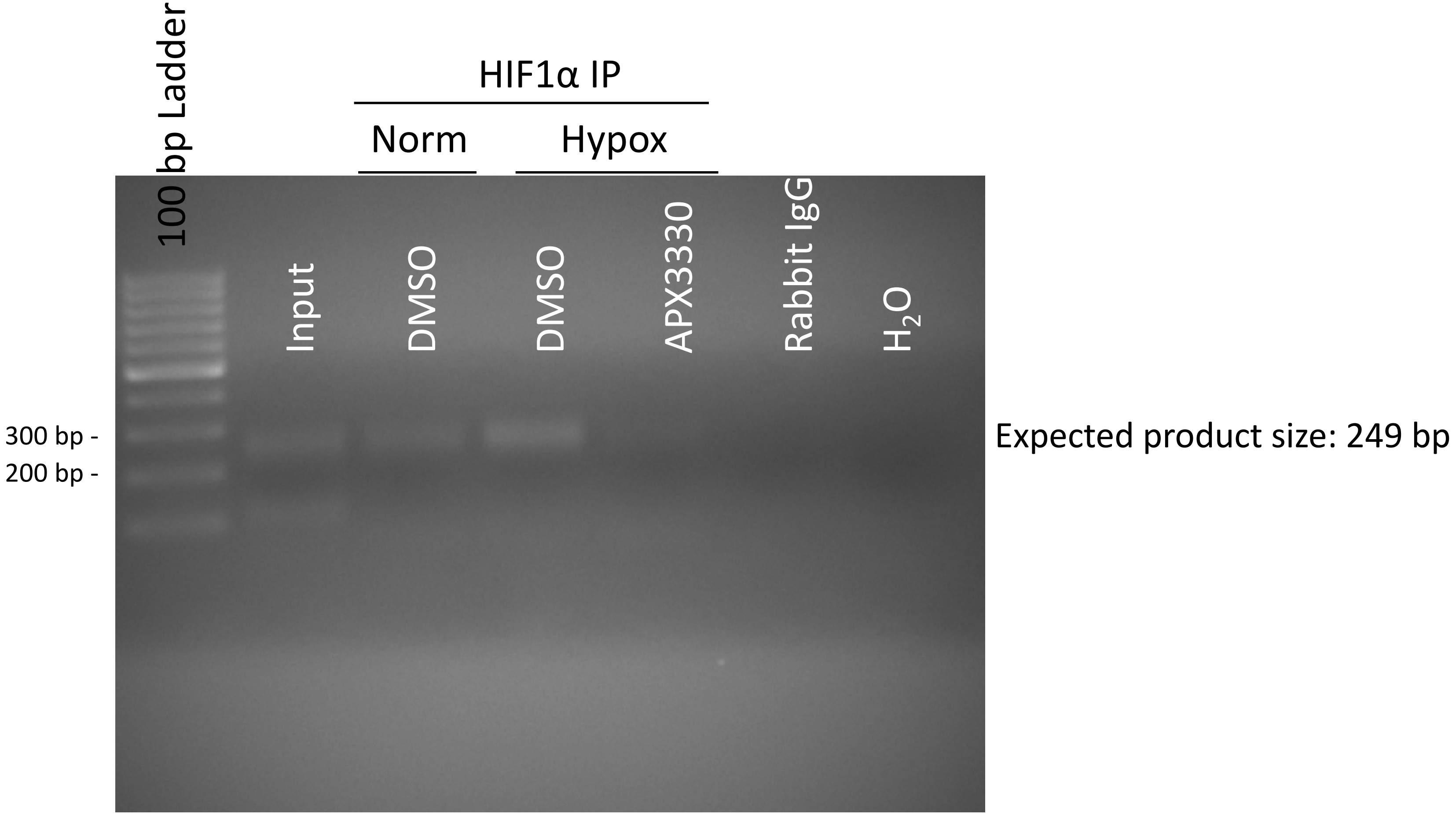

Supplemental Figure S6B. Full blot for Figure 2D: CA9

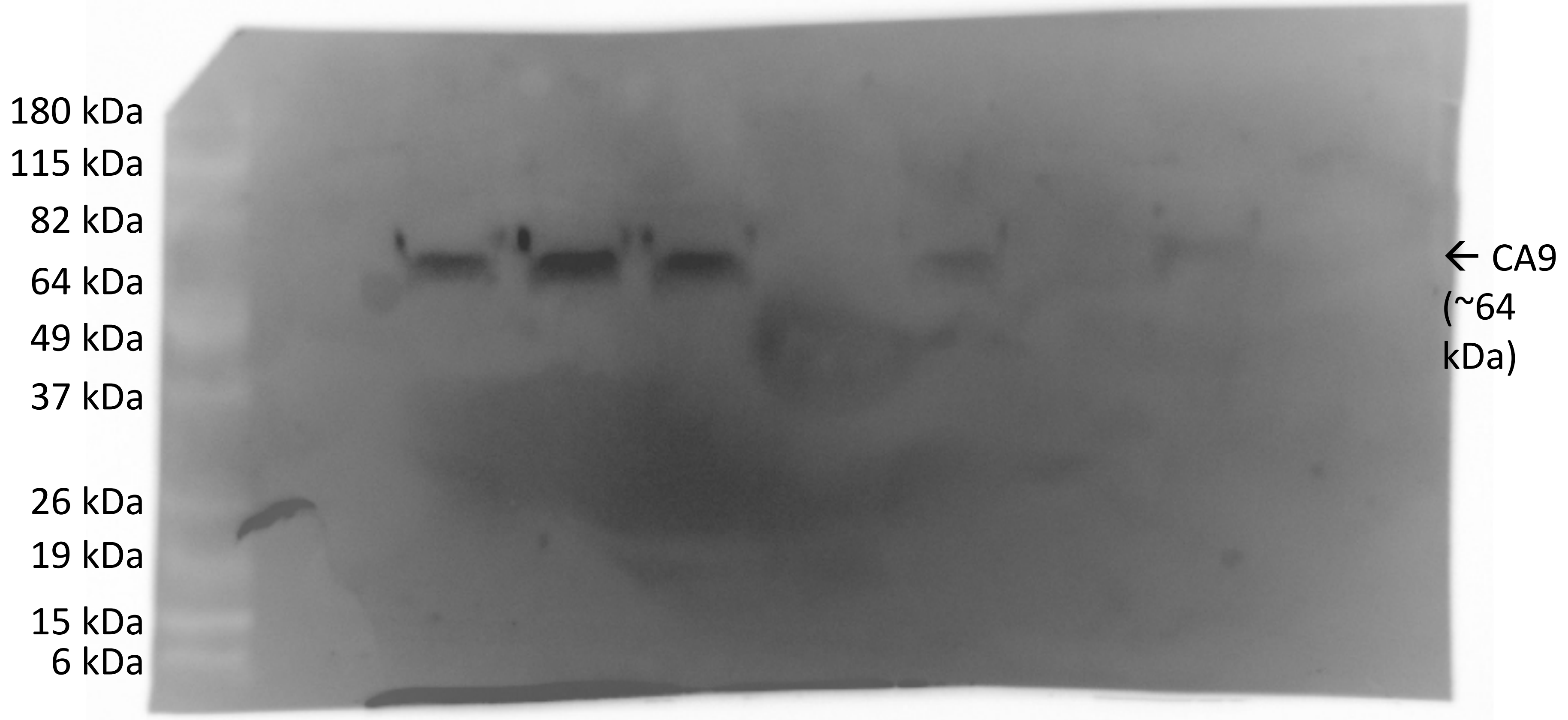

Supplemental Figure S6C. Full blot for Figure 2D: APE1/Ref-1

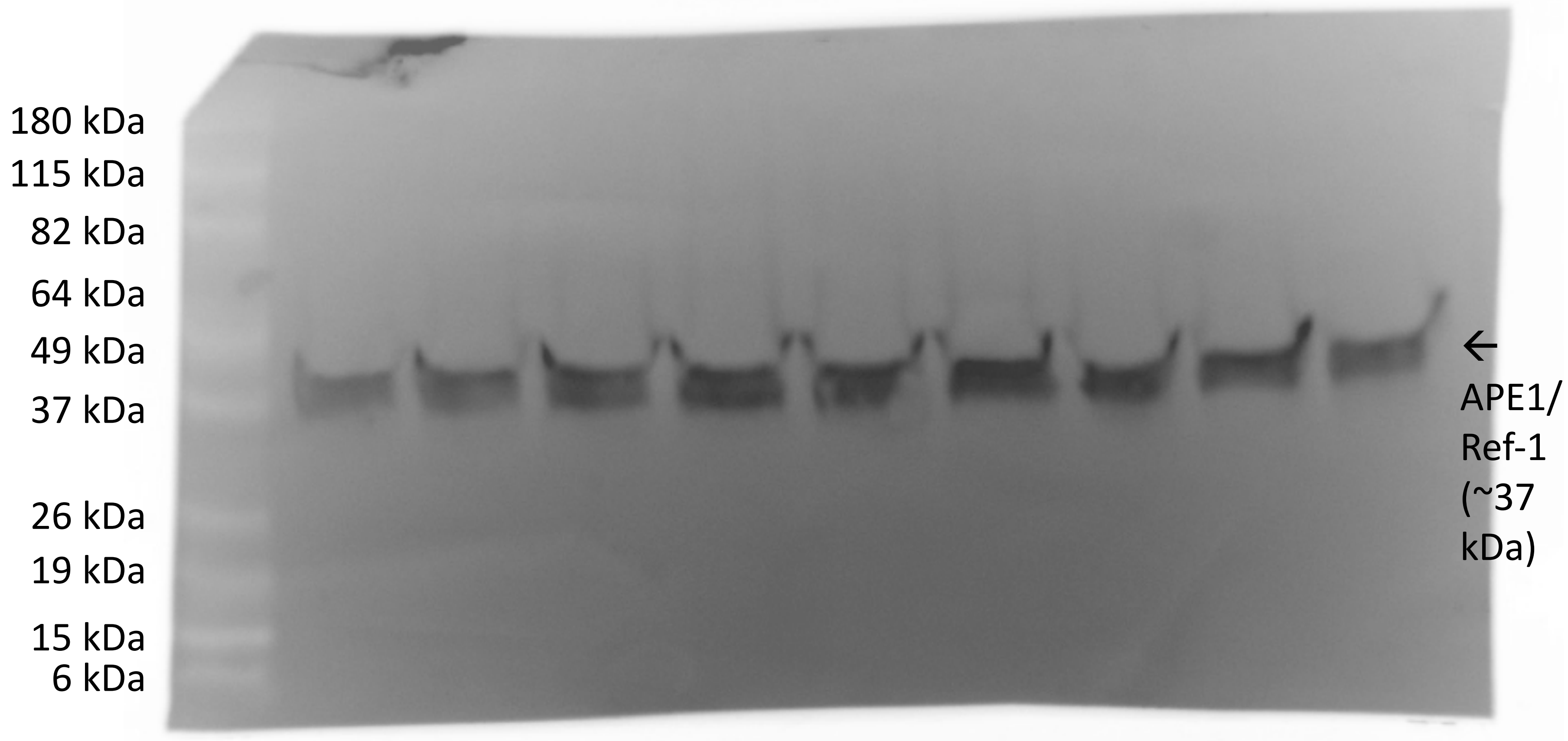

Supplemental Figure S6D. Full blot for Figure 2D: Vinculin

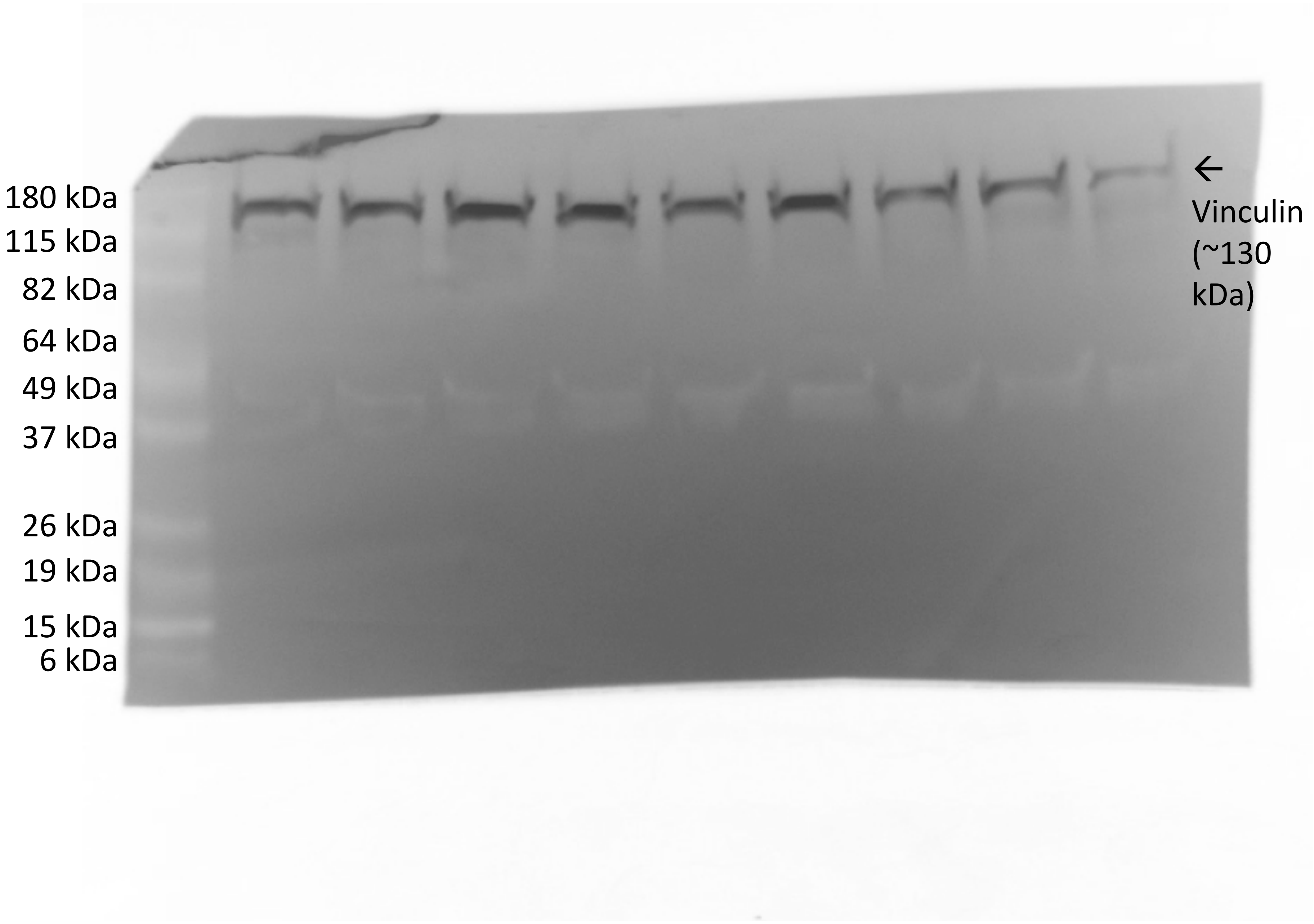

Supplemental Figure S7A. Full blot for Figure 2E: CA9

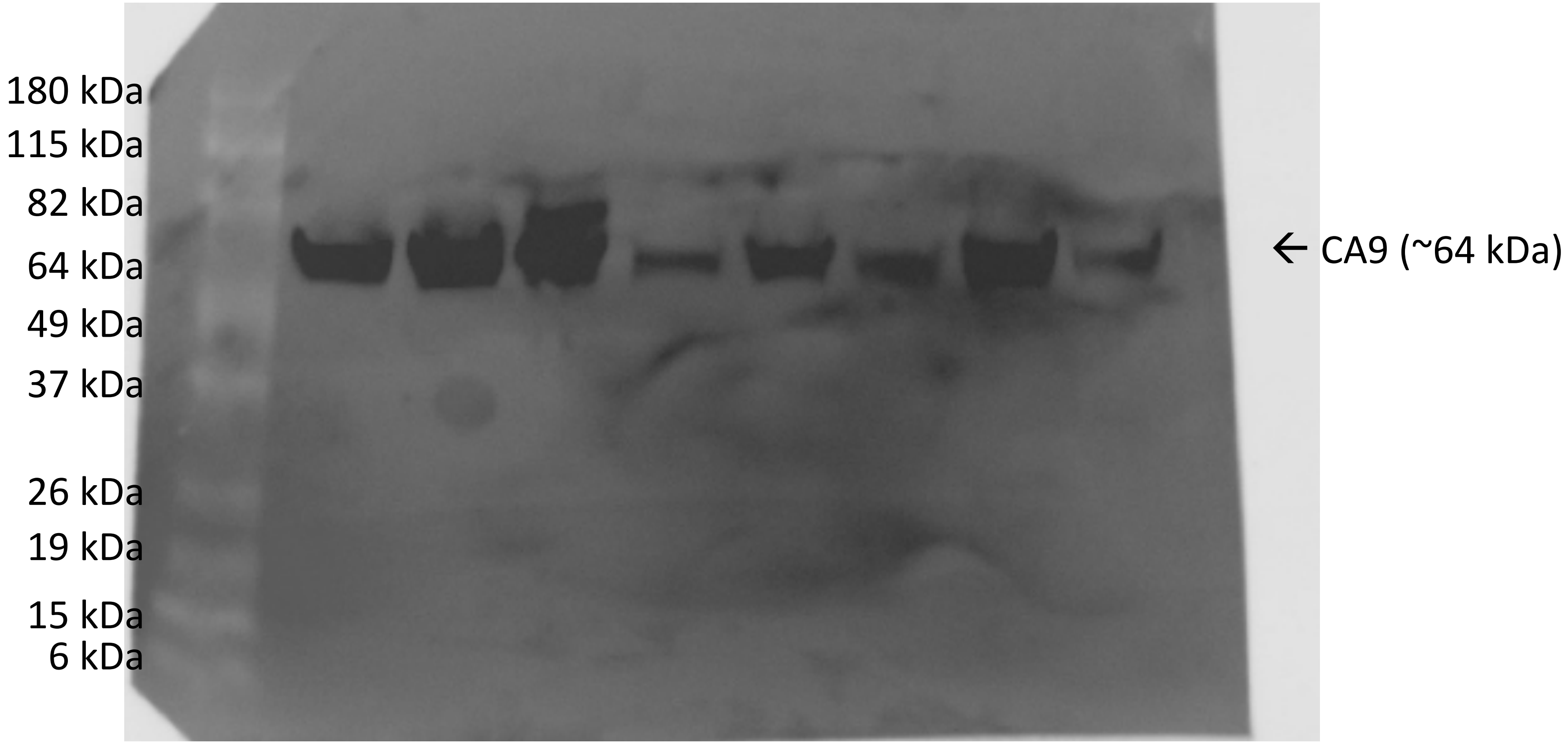

Supplemental Figure S7B. Full blot for Figure 2E: APE1/Ref-1

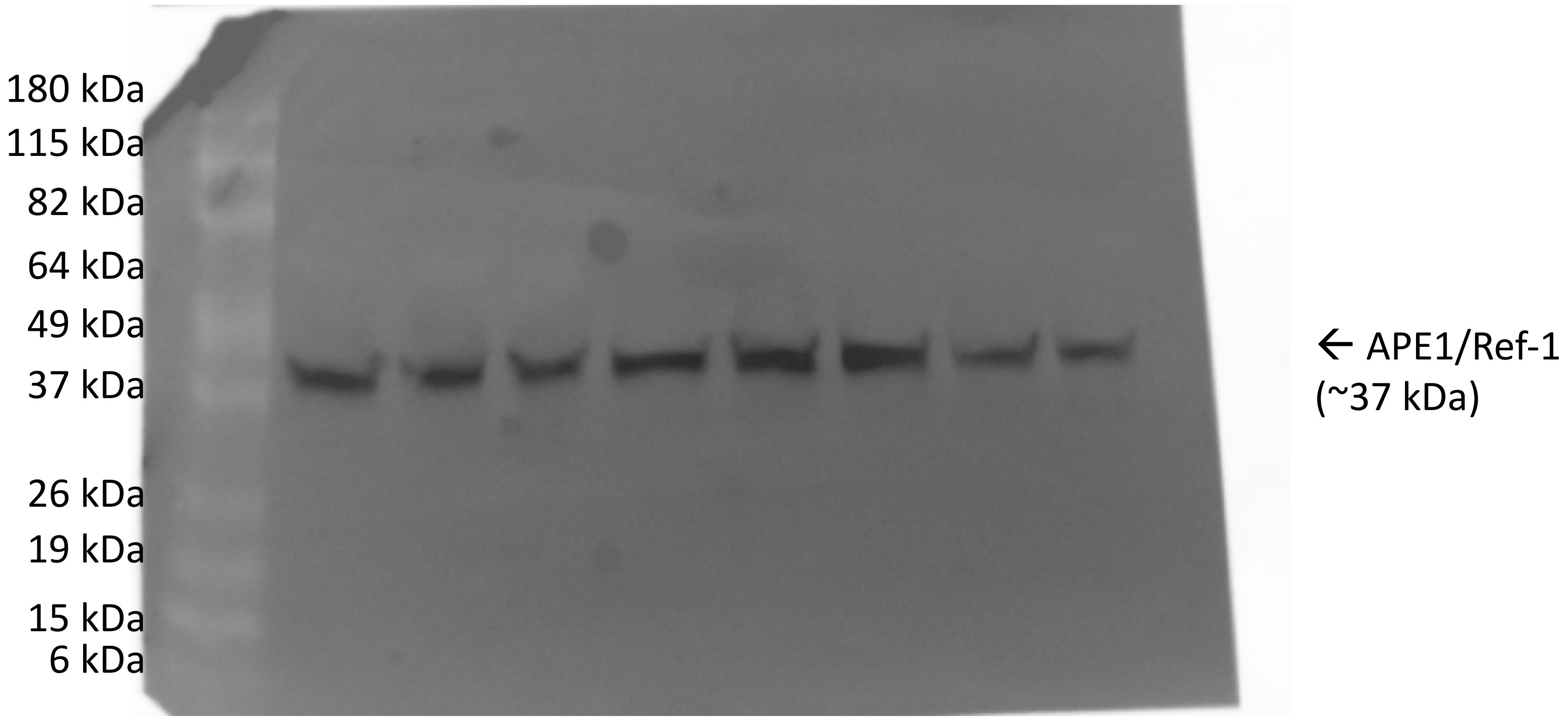

Supplemental Figure S7C. Full blot for Figure 2E: Vinculin

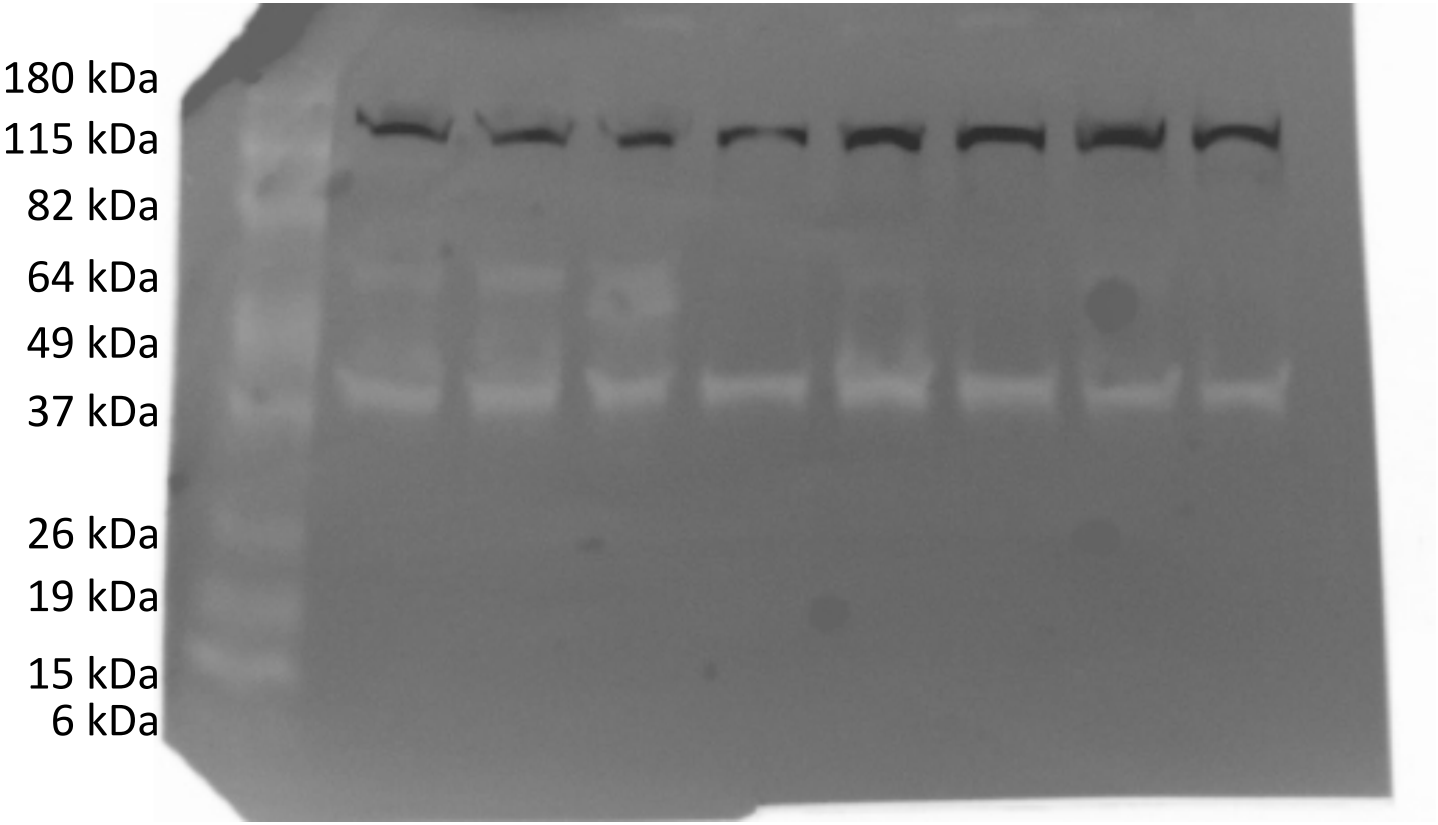

Supplement: Supplementary file 1 — Supplemental information and figures [file 41598_2018_32034_MOESM1_ESM.pdf]
